# Evaluating Clinical and Sociodemographic Risk for Symptom Burden Associated Interference With Daily Functioning in the Primary Brain Tumor Patient Population

**DOI:** 10.1002/cam4.70682

**Published:** 2025-03-07

**Authors:** Bennett A. McIver, Tara S. Davis, Kimberly Reinhart, Elizabeth Vera, Alvina Acquaye‐Mallory, Anna Choi, Tricia Kunst, Morgan Johnson, Ewa Grajkowska, Hope Miller, Jennifer Reyes, Mark R. Gilbert, Terri S. Armstrong, Michelle L. Wright

**Affiliations:** ^1^ Neuro‐Oncology Branch National Cancer Institute, National Institutes of Health Bethesda Maryland USA

**Keywords:** activity‐related interference, functional interference, mood‐related interference, primary brain tumors

## Abstract

**Introduction:**

Symptom burden associated with interference in daily functioning is worse in those with progression or higher‐grade glial tumors. This exploratory study aims to identify factors associated with its severity in a diverse cross‐sectional cohort of 566 brain tumor patients enrolled in a natural history study (NCT03251989, PI: T.S. Armstrong).

**Methods:**

Sociodemographic and clinical data and self‐reported activity‐related interference (work, general activity, walking), mood‐related interference (relations with others, enjoyment of life, mood) were reported via the MD Anderson Symptom Inventory‐Brain Tumor. Activity and mood‐related interference mean scores ≥ 2 were categorized as moderate–severe. Logistic regression assessed univariate associations with moderate–severe interference. Characteristics significant in the univariate analysis were included in a multivariable analysis.

**Results:**

This patient sample had a median age of 48 years (18–85), was mostly male (57%), with a high‐grade tumor (73%), glioblastoma (39%), and tumor recurrence (49%). Risk factors for moderate–severe activity‐related interference included: ≥ 2 surgeries (OR = 1.64, 95% CI [1.10, 2.44], *p* = 0.015), ependymoma (OR = 2.59, 95% CI [1.21–5.53], *p* = 0.014), and childhood in a rural area (OR = 1.74, 95% CI [1.15–2.63] *p* = 0.009). Risk factors for moderate–severe mood‐related interference included tumor progression (OR = 2.02, 95% CI [1.21–3.36], *p* = 0.009).

**Conclusion:**

Patient reported interference with daily physical functioning is associated with sociodemographic and disease‐related characteristics and notably worse mood‐related interference in those with progression. Future studies should include social determinants of health and change over time to identify and plan interventions for those at risk.

## Graphical abstract

Activity and mood‐related interference is common in primary brain tumor patients. Severity is associated with both clinical factors (diagnosis, number of surgeries) and social determinants of health (rural disparities).

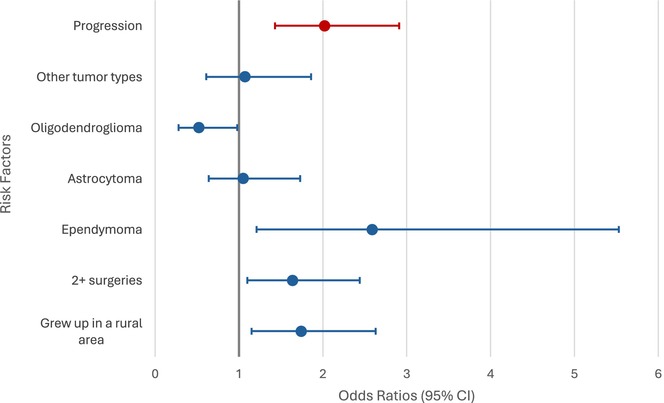

## 1 Introduction

Patients with cancer may face a multitude of physical and psychosocial symptoms resulting from both diagnosis and treatment [1, 2]. Previous studies have shown that symptoms seldom occur in isolation, and both the number and severity of symptoms can impact health outcomes such as reduced mobility, quality of life, and physical functioning [3, 4]. Measuring the individual's perception of symptom‐associated interference with daily life is essential for understanding a patient's quality of life during the illness trajectory [5].

Although symptoms that result in interference are seen across all cancer types, primary brain tumors have a high propensity for causing severe symptoms and functional impairment that interfere with daily life due to the nature of their location in the brain [6, 7]. This occurs even with benign or small tumors located in eloquent regions leading to significant neurologic or cognitive symptoms [8]. Additionally, core symptoms associated with systemic cancer and its treatment occur with similar frequency in these patients [9, 10, 11]. Even in those considered long‐term survivors, nearly half have reported three or more severe physical symptoms and nearly a quarter reported significant mood‐related symptoms that interfere with daily life [12]. Past studies have examined the impact of primary brain tumors on many facets of quality of life, thus elucidating the physical, psychological, and social components of disease burden [13, 14, 15]. Specifically, studies have found that primary brain tumors may cause severe physical functional decline in patients [7, 13, 16]. A decline in physical function may contribute to difficulty working [7], completing everyday activities [17, 18], and walking [16, 19]. Additionally, the symptoms associated with primary brain tumors have been shown to interfere with patients' relationships with others [20], emotional well‐being [21], and mood [19, 21, 22].

Understanding the underlying risk factors for patient report of interference with daily functioning is essential for developing targeted interventions to prevent or manage its occurrence [11]. In the broader cancer patient population, social determinants of health (SDOH), including socioeconomic status, access to care, and living conditions, likely contribute to the severity of certain symptoms such as fatigue, sleep dysregulation, and depression [23, 24]. A review of the relationship of social determinants of health in the primary brain tumor patient population with mood and cognitive symptoms revealed that residing in a rural area may contribute to mood‐related symptoms, and individuals with higher educational attainment may experience fewer neurocognitive symptoms [25]. To our knowledge, clinical and demographic factors, including key social determinants of health that may disproportionately put patients at risk of experiencing greater symptom‐related interference among primary brain tumor patients, have not been previously explored among primary brain tumor patients. The purpose of this exploratory study was to identify clinical and sociodemographic factors associated with more severe activity and mood‐related interference in a primary brain tumor population.

## 2 Materials & Methods

### 2.1 Patient Sample

Patients were identified from the National Cancer Institute's Natural History Study (NCT02851706; PI: Terri S. Armstrong). Included in this analysis were English‐speaking patients diagnosed with primary brain tumors enrolled between September 2016 and February 2020. The Natural History Study (NOB‐NHS) is an observational, longitudinal study that collects demographic, clinical, physical, and patient‐reported outcome measures (PROs) from patients diagnosed with central nervous system tumors throughout the course of their disease. The NHS was approved by the Institutional Review Board, and written, informed consent was obtained from each participant. The questionnaire used to collect early life data was acquired from the Risk and Outcomes Survey administered at the study entry timepoint for the Natural History Study. The Risk and Outcomes Survey and the questions regarding early life experiences were adapted from the Glioma Outcomes Project. The language used in the questions on the Risk and Outcomes Survey was directly adapted from the language in the Glioma Outcomes Project, which was used in prior studies of the primary brain tumor population [26, 27, 28].

### 2.2 Measures

Data from the NHS collected between September 2016 and February 2020 was used in this analysis. Patients completed a demographic questionnaire that included information on age, sex, race, ethnicity, employment, education level, income, and marital status. This questionnaire was completed either in clinic or virtually, prior to receiving the results of their most recent MRI scan. Social determinants of health and early life exposure, including family income level growing up, growing up in a rural area, and having enough to eat growing up, were also assessed; however, 79 of the 566 patients in this analysis did not complete this questionnaire. The M. D. Anderson Symptom Inventory—Brain Tumor Module (MDASI‐BT) was administered at study entry. The MDASI‐BT is an assessment of symptom burden and interference, which includes 22 symptoms and 6 interference items rated from 0 (not present/did not interfere) to 10 (as worse as you can imagine/interfered completely) over the past 24 h [7]. Interference of symptoms with daily life is divided into two subscales to capture the impact of symptom‐related functional impairment: activity‐related functional interference (WAW), consisting of work, general activity, and walking, and mood‐related interference (REM), consisting of relations with other people, enjoyment of life, and mood [19, 29, 30, 31]. Interference items rated 2 or higher are categorized as moderate–severe [32, 33].

Clinical and treatment information were collected using a series of standardized items collected electronically by trained clinical staff at the time of clinical evaluation. This included months from diagnosis to study entry, tumor diagnosis, tumor grade, tumor location, tumor recurrence, active treatment status, previous treatment, number of recurrences, current disease status based on most recent imaging, medications, surgeries, and radiation history. Tumor recurrence indicates that a previously treated tumor has returned, and it is reported as the number of times the tumor has returned. Progression status indicates whether a patient's tumor has grown since a prior scan, which is assessed by asking a patient to answer “yes or no” regarding whether their tumor has progressed. The Karnofsky Performance Status (KPS) was rated by trained clinical providers at the time of study entry, with scores ranging from 0 to 100 to indicate the patient's ability to carry out activities of daily living [34, 35].

### 2.3 Statistical Analysis

Statistical analyses were conducted with IBM SPSS Version 25.0.41 [36]. Descriptive statistics characterized the patient sample and provided PRO summary scores at study entry. Progression status was determined according to the MRI results at the study entry time point. KPS was dichotomized based on score into good (≥ 90) and poor (≤ 80) based on findings that KPS scores ≤ 80 have a greater symptom burden and are clinically relevant in that scores of 80 or below are associated with tumor progression [7, 11, 37].

Logistic regression was first used to identify univariate associations with moderate–severe activity‐ and mood‐related interference among demographic and clinical characteristics. An adjustment for multiple comparisons was applied via the Bonferroni‐Holm correction, with the new significance threshold set to *p* < 0.002. Demographic and clinical characteristics that were significant at the *p* < 0.1 level in the univariate analysis were included in a subsequent multivariable analysis with backwards selection set at *p* < 0.05. Although KPS, employment, and income were evaluated for association with symptom‐related interference, they were not included in the multivariable model due to the following reasons: KPS and employment could be interpreted as a result of interference rather than a risk factor for interference, and income had a high degree of missing data due to its later inclusion on the study demographic questionnaire. Multivariable regression was chosen due to the numerous potential confounding variables. Time to diagnosis was forced into the model to further explain the relationships between tumor diagnosis and significant interference. This analysis is an exploratory analysis, and thus, it is not typical to perform a power calculation. However, a post hoc power analysis was performed for this sample. With 80% power, an alpha of 0.05, and a total sample of 566 participants, the smallest odds ratio we can reliably detect is 1.6. This is based on using a reference proportion of 42%, which is based on our previously published findings [12].

## 3 Results

### 3.1 Sample Characteristics

Five hundred sixty‐six patients with primary brain tumors were included in this study, with complete characteristics presented in Table 1. More men (57%) than women were included in the sample, with a median age of 48 years (range 18–85). The patients were also primarily white (81%), non‐Hispanic (86%), married or living with a partner (69%), nearly half were employed (49%), and less than a quarter had an income between $50,000–$149,999 per year (22%). Most patients had high‐grade tumors (73%) and the most common diagnosis was glioblastoma (39%). Slightly more than half had a good KPS (53%), had undergone only one surgery (61%) and radiation treatment (64%), and a little more than half had no prior tumor recurrence (51%). Most did not have disease progression on imaging (79%) performed at study entry. Out of the 489 patients who completed the early life exposures questionnaire, the majority of patients categorized their income level while growing up as “middle income” (65%) and stated that they grew up in an urban area (66%), and a small portion of patients reported that they did not have enough to eat when growing up (6%).

**TABLE 1:** Patient demographics and clinical characteristics (*N* = 566).

Characteristic	Results	
Age	Median (range)	48 (18–85)
	Mean (SD)	48 (14)
Time from diagnosis	Median (range)	21 (0–376)
	Mean (SD)	56 (68)

		*N* (%)
Sex	Female	245 (43)
	Male	321 (57)
Race	Asian	30 (5)
	Black/African American	37 (7)
	White	458 (81)
	Native Hawaiian/Pacific Islander	3 (0.5)
	Other	8 (1)
	Unknown	30 (5)
Ethnicity	Not Hispanic or Latino	488 (86)
	Hispanic or Latino	53 (9)
	Unknown	25 (4)
Highest education level	≤ High school education	76 (13)
	Associate's degree/any college	93 (16)
	Bachelor's degree	183 (32)
	Advanced degree	195 (34)
	Unknown	19 (3)
Marital status	Married/Living with a partner	390 (69)
	Single	116 (21)
	Divorced/Separated/Widowed	53 (9)
	Unknown	7 (1)
Employment status	Employed	277 (49)
	Retired	106 (19)
	On disability	62 (11)
	Unemployed	103 (18)
	Unknown	18 (3)
Income	< $49,999	69 (12)
	$50,000–$149,999	125 (22)
	More than $150,000	60 (11)
	Prefer not to answer	45 (8)
	Unknown	267 (47)
Tumor grade	Low grade (1/2)	143 (25)
	High grade (3/4)	416 (73)
	Unknown	7 (1)
Top tumor diagnosis	Astrocytoma	124 (22)
	Ependymoma	46 (8)
	Glioblastoma	220 (39)
	Oligodendroglioma	72 (13)
	Other tumor type	104 (18)
KPS	Poor (50–80)	260 (46)
	Good (90–100)	299 (53)
	Unknown	7 (1)
Number of surgeries	1	344 (61)
	≥ 2	222 (39)
Number of radiation treatments	0	124 (22)
	1	365 (64)
	≥ 2	77 (14)
Recurrence	No	290 (51)
	Yes	276 (49)
Progression	No progression	448 (79)
	Progression	118 (21)
Income level*	Poor	17 (3)
	Low income	81 (17)
	Middle income	320 (65)
	Well off	50 (10)
	Unknown	21 (4)
Growing up in a rural area*	No	322 (66)
	Yes	144 (29)
	Unknown	23 (5)
Having enough to eat growing up*	Yes	436 (89)
	No	31 (6)
	Unknown	22 (4)

*Note:* Categories marked by an * display data from a questionnaire completed by only 489 out of the 566 patients.

### 3.2 Interference With Daily Life

Of the total sample, nearly half (48%) of participants reported moderate–severe activity‐related interference, with 39% reporting interference with walking, 50% with general activity, and 51% with work. Additionally, nearly half (48%) of the patients reported experiencing moderate–severe mood‐related interference, with 38% reporting moderate–severe interference with relationships, 46% reporting interference with enjoyment of life, and 50% reporting interference with mood. Finally, 230 of the participants (41%) reported both activity and mood‐related interference.

### 3.3 Prediction of Risk Factors of Activity‐Related Interference (WAW)

Univariate analyses of moderate–severe activity‐related interference with SDOH and demographic characteristics indicate employment status to be a significant predictor of activity‐related interference, when adjusting for multiple comparisons. Specifically, patients who were unemployed (OR 3.81, 95% CI [2.34–6.19]), on disability (OR 3.20, 95% CI [1.79–5.71]) or retired (OR 1.77, 95% CI [1.12–2.78]) had greater odds of having moderate–severe activity‐related interference compared to their employed counterparts, with no other demographic factors associated with risk (Table 2). Clinical characteristics associated with higher odds of moderate–severe activity‐related interference included having a poor KPS (OR 3.61, 95% CI [2.55–5.12]), and patients who received one radiation treatment were more likely to have moderate–severe activity‐related interference than those who had no radiation (OR 1.68, 95% CI [1.11–2.56]) and those with two or more radiation treatments were more likely (OR 2.87, 95% CI [1.59–5.17]) compared to those who had not received radiation (Table 2).

**TABLE 2:** Univariate associations between moderate‐severe WAW and demographic and clinical characteristics (*N* = 566).

	Predictor		Odds ratio	95% CI	*p*‐value
**Demographic characteristics**	Age		1.00	0.99–1.02	0.473
	Sex	Male	1.11	0.8–1.55	0.538
		Female	Reference		
	Race				0.854
		White	Reference		
		Black	1.09	0.56–2.13	0.795
		Asian	0.91	0.43–1.90	0.794
		Other race	1.24	0.37–4.13	0.723
	Ethnicity	Hispanic or Latino	1.52	0.85–2.70	0.155
		Not Hispanic or Latino	Reference		
	Education				0.011
		≤ High school education	2.03	1.18–3.51	0.011
		Associate's degree/Any college	1.30	0.79–2.14	0.296
		Bachelor's degree	0.89	0.59–1.33	0.569
		Advanced degree	Reference		
	**Employment status**				**< 0.001**
		**Employed**	**Reference**		
		**Retired**	**1.77**	**1.12–2.78**	**0.014**
		**On disability**	**3.20**	**1.79–5.71**	**< 0.001**
		**Unemployed**	**3.81**	**2.34–6.19**	**< 0.001**
	Family income level when growing up				0.924
		Poor	Reference		
		Low income	1.62	0.56–4.67	0.374
		Middle income	1.18	0.44–3.19	0.739
		Well off	1.22	0.40–3.71	0.730
	Income				0.107
		Less than $49,999	2.62	1.28–5.35	0.008
		$50,000–$149,999	1.61	0.87–3.00	0.132
		More than $150,000	Reference		
	Grew up in a rural area	No	Reference		
		Yes	1.87	1.24–2.84	0.003
	Did not have enough to eat growing up	No	Reference		
		Yes	1.56	0.85–2.86	0.153
**Clinical characteristics**					
	Top tumor diagnoses				0.317
		Glioblastoma	Reference		
		Astrocytoma	1.00	0.64–1.55	1.000
		Ependymoma	2.07	1.06–4.04	0.034
		Oligodendroglioma	0.50	0.29–0.87	0.015
		Other tumor types	1.08	0.68–1.72	0.747
	Tumor grade	Low grade (1/2)	Reference		
		High grade (3/4)	1.05	0.72–1.54	0.790
	Number of surgeries	1	Reference		
		≥ 2	1.55	1.11–2.18	0.011
	**Number of radiation treatments**				**< 0.001**
		**0**	**Reference**		
		**1**	**1.68**	**1.11–2.56**	**0.014**
		**≥ 2**	**2.87**	**1.59–5.17**	**< 0.001**
	Number of treatments				0.147
		0	Reference		
		1	1.07	0.72–1.57	0.751
		≥ 2	1.37	0.91–2.07	0.136
	Active treatment at the time of visit	No	Reference		
		Yes	1.47	0.97–2.22	0.066
	Progression status	No progression	Reference		
		Progression	1.87	1.24–2.84	0.003
	Tumor recurrence	No	Reference		
		Yes	1.57	1.13–2.19	0.007
	**KPS**	**Poor (50–80)**	**3.61**	**2.55–5.12**	**< 0.001**
		**Good (90–100)**	**Reference**		
	Charlson Comorbidity Index Score		0.91	0.79–1.05	0.210
	Charlson Comorbidity Index score categories				0.172
		0	Reference		
		1	1.07	0.64–1.80	0.784
		2 +	0.71	0.45–1.12	0.141
	BMI	Mean (SD)	0.99	0.96–1.02	0.482
	BMI Categories				0.995
		Underweight/Normal Weight (< 25 kg/m^2^)	Reference		
		Overweight (25–29.99 kg/m^2^)	0.87	0.58–1.29	0.477
		Obese (≥ 30 kg/m^2^)	1.03	0.68–1.57	0.892
	Weight (kg) year prior to diagnosis		1.00	0.99–1.01	0.658
	Typical adult weight (kg)		1.00	0.99–1.01	0.548
	Completed light activity in the past 10 years				0.870
		< 1 h per week	Reference		
		1–3 h per week	1.20	0.67–2.14	0.549
		4–7 h per week	1.21	0.69–2.13	0.509
		> 7 h per week	0.98	0.56–1.71	0.937
	Completed moderate/vigorous activity in the past 10 years				0.508
		< 1 h per week	Reference		
		1–3 h per week	0.93	0.56–1.55	0.774
		4–7 h per week	0.93	0.55–1.58	0.792
		> 7 h per week	0.81	0.46–1.43	0.471
	Smoking status	Never/former smoker	Reference		
		Current smoker	0.96	0.42–2.19	0.921

*Note:* Bolded categories indicate a significant *p*‐value when adjusted for multiple comparisons (*p* < 0.002).

Abbreviations: CI, confidence interval; KPS, Karnofsky Performance Status.

Variables with *p* < 0.10 in the univariate analyses included in the subsequent multivariate analyses as potential predictors of moderate–severe activity‐related interference were time from diagnosis, education, growing up in a rural area, tumor diagnosis, tumor grade, surgeries, radiation treatments, on treatment at the time of visit, progression status, and tumor recurrence. According to the final model of the multivariate analysis, growing up in a rural area, tumor diagnostic name, and number of surgeries were significant risk factors for activity‐related interference, as described in Table 3. Patients who experienced growing up in a rural area had 1.74 greater odds (OR 1.74, 95% CI [1.15–2.63]), those who had 2 or more surgeries had 1.64 times the odds compared to those who only had 1 surgery (OR 1.64, 95% CI [1.10–2.44]), whereas patients diagnosed with a brain ependymoma had the highest chance of reporting a moderate–severe interference with activity, with 2.59 times increased odds of moderate–severe activity‐related interference compared to those with glioblastoma (OR 2.59, 95% CI [1.21–5.53]). However, glioblastoma patients had greater odds of having moderate–severe activity‐related interference compared to those with an oligodendroglioma, who had 0.52 times the odds of moderate–severe activity‐related interference (OR 0.52, 95% CI [0.28–0.98]).

**TABLE 3:** Multivariate associations between moderate‐severe WAW and demographic and clinical characteristics (*N* = 566).

	Predictor		Full Model			Final Model		
			Odds ratios	95% CI	*p*‐value	Odds ratio	95% CI	*p*‐value
**Demographic characteristics**	Time from diagnosis		1.00	0.99–1.00	0.036			
	Education				0.238			
		≤ High school education	1.35	0.69–2.63	0.377			
		Any college	1.06	0.58–1.94	0.841			
		Bachelor's degree	0.72	0.45–1.15	0.164			
		Advanced degree	Ref.					
	Grew up in a rural area				0.061			0.009
		No	Ref.			Ref.		
		Yes	1.52	0.98–2.35	0.061	1.74	1.15–2.63	0.009
**Clinical characteristics**	Top tumor diagnosis				0.043			0.012
		Glioblastoma	Ref.			Ref.		
		Astrocytoma	1.31	0.72–2.37	0.376	1.05	0.64–1.73	0.853
		Ependymoma	2.86	1.25–6.54	0.013	2.59	1.21–5.55	0.014
		Oligodendroglioma	0.72	0.35–1.46	0.357	0.52	0.28–0.98	0.042
		Other type	1.12	0.59–2.12	0.725	1.07	0.61–1.86	0.816
	Tumor grade				0.172			
		Low grade (1/2)	1.49	0.84–2.62	0.172			
		High grade (3/4)	Ref.					
	Number of surgeries				0.076			0.015
		1	Ref.			Ref.		
		≥ 2	1.57	0.95–2.58	0.076	1.64	1.10–2.44	0.015
	Number of radiation treatment				0.235			
		0	Ref.					
		1	1.45	0.81–2.59	0.216			
		≥ 2	2.15	0.88–5.26	0.092			
	Active treatment at the time of visit				0.132			
		No	Ref.					
		Yes	1.50	0.89–2.52	0.132			
	Progression status				0.535			
		No progression	Ref.					
		Progression	1.22	0.65–2.28	0.535			
	Tumor recurrence				0.580			
		No	Ref.					
		Yes	1.17	0.67–2.06	0.580			

Abbreviations: CI, confidence interval; Ref, reference group.

### 3.4 Prediction of Risk Factors of Mood‐Related Interference (REM)

**FIGURE 1:**
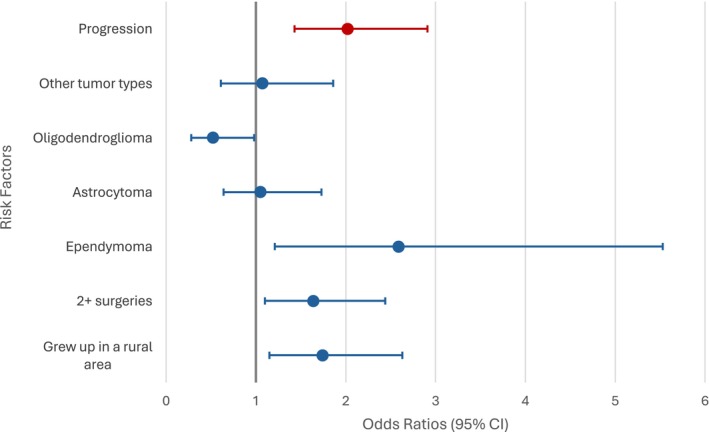
Multivariate analysis of variables associated with moderate–severe activity and mood‐related interference. CI, confidence interval; red odds ratio and confidence interval indicative of risk factors for mood‐related interference; blue odds ratios and confidence intervals indicative of risk factors for activity‐related interference; No progression used as a reference group for progression status, Glioblastoma used as a reference group for tumor diagnosis, 1 surgery used as a reference group for number of surgeries, Did not grow up in a rural area used as a reference group for growing up in a rural area.

Univariate analysis indicates that employment status is also the most significant SDOH or demographic risk factor for moderate–severe mood‐related interference, as shown in Table 4. Patients who were on disability (OR 2.83, 95% CI [1.61–4.97]) or unemployed (OR 3.65, 95% CI [2.27–5.86]) had increased odds of having moderate–severe mood‐related interference compared to patients who were employed, while being retired was not found to be significant. Tumor progression at the time of assessment and KPS were found to be significant clinical risk factors for moderate–severe mood‐related interference, with those who experienced progression (OR 2.26, 95% CI [1.50–3.41]) and those with a poor KPS (OR 2.84, 95% CI [2.01, 4.01]) having a higher odd of moderate–severe mood‐related interference. Variables with *p* < 0.10 in the univariate analyses included in the subsequent multivariate analyses included not having enough to eat growing up, number of surgeries, number of radiation treatments, progression status, and tumor recurrence, as well as the additions of time to diagnosis, education level, growing up in a rural area, and tumor type. According to the final model of the multivariate analysis as indicated in Table 5 and Figure 1, progression status according to the MRI results at the time of study entry was the only variable found to be significantly associated with moderate–severe mood‐related interference, with patients experiencing tumor progression (OR 2.02, 95% CI [1.21–3.36]) having greater odds of moderate–severe mood‐related interference compared to those who did not have tumor progression.

**TABLE 4:** Univariate associations between moderate‐severe REM and demographic and clinical characteristics (*N* = 566).

	Predictor		Odds ratio	95% CI	*p*‐value
**Demographic characteristics**	Age		1.00	0.98–1.01	0.459
	Sex	Male	1.04	0.74–1.46	0.821
		Female	Reference		
	Race				0.825
		White	Reference		
		Black	0.94	0.47–1.85	0.850
		Asian	0.92	0.43–1.95	0.818
		Other race	1.14	0.34–3.80	0.826
	Ethnicity	Hispanic or Latino	1.15	0.65–2.04	0.626
		Not Hispanic or Latino	Reference		
	Education				0.143
		≤ High school education	1.56	0.92–2.66	0.100
		Associate's degree/Any college	1.06	0.64–1.75	0.813
		Bachelor's degree	0.93	0.62–1.41	0.745
		Advanced degree	Reference		
	**Employment status**				**< 0.001**
		**Employed**	**Reference**		
		**Retired**	**1.24**	**0.78–1.97**	**0.368**
		**On disability**	**2.83**	**1.61–4.97**	**< 0.001**
		**Unemployed**	**3.65**	**2.27–5.86**	**< 0.001**
	Family Income level when growing up				0.211
		Poor	Reference		
		Low income	0.90	0.32–2.57	0.844
		Middle income	0.69	0.26–1.85	0.463
		Well off	0.63	0.21–1.93	0.421
	Income				0.317
		Less than $49.999	1.59	0.79–3.19	0.193
		$50,000–$149,999	0.99	0.53–1.84	0.980
		More than $150,000	Reference		
	Grew up in a rural area	No	Reference		
		Yes	1.31	0.88–1.95	0.186
	Did not have enough to eat growing up	No	Reference		
		Yes	2.23	1.07–4.67	0.033
**Clinical characteristics**					
	Top tumor diagnosis				0.312
		Glioblastoma	Reference		
		Astrocytoma	0.99	0.64–1.54	0.958
		Ependymoma	0.92	0.49–1.75	0.807
		Oligodendroglioma	0.46	0.26–0.82	0.009
		Other tumor type	0.92	0.57–1.46	0.712
	Tumor grade	Low grade (1/2)	Reference		
		High grade (3/4)	1.08	0.73–1.58	0.712
	Number of surgeries	1	Reference		
		≥ 2	1.33	0.95–1.87	0.099
	Number of radiation treatments				0.031
		0	Reference		
		1	1.47	0.96–2.25	0.075
		≥ 2	1.84	1.03–3.28	0.040
	Number of treatments				0.458
		0	Reference		
		1	1.09	0.74–1.62	0.656
		≥ 2	1.17	0.77–1.78	0.463
	Active treatment at the time of visit	No	Reference		
		Yes	0.98	0.65–1.49	0.941
	**Progression status**	**No progression**	**Reference**		
		**Progression**	**2.26**	**1.50–3.41**	**< 0.001**
	Tumor recurrence	No	Reference		
		Yes	1.57	1.12–2.19	0.009
	**KPS**	**Poor (50–80)**	**2.84**	**2.01–4.01**	**< 0.001**
		**Good (90–100)**	**Reference**		
	Charlson Comorbidity Index Score		0.85	0.74–0.99	0.034
	Charlson Comorbidity Index score categories				0.017
		0	Reference		
		1	1.13	0.68–1.87	0.647
		2 +	0.54	0.33–0.86	0.010
	BMI	Mean (SD)	0.99	0.96–1.02	0.467
	BMI categories				0.982
		Underweight/normal weight (< 25 kg/m^2^)	Reference		
		Overweight (25–29.99 kg/m^2^)	0.95	0.64–1.42	0.813
		Obese (≥ 30 kg/m^2^)	0.99	0.65–1.52	0.966
	Weight (kg) year prior to diagnosis		1.00	0.99–1.01	0.557
	Typical adult weight (kg)		1.00	0.99–1.01	0.525
	Completed light activity in the past 10 years				0.315
		< 1 h per week	Reference		
		1–3 h per week	1.06	0.59–1.91	0.835
		4–7 h per week	0.63	0.35–1.13	0.119
		> 7 h per week	0.86	0.49–1.52	0.613
	Completed moderate/vigorous activity in the past 10 years				0.401
		< 1 h per week	Reference		
		1–3 h per week	0.94	0.56–1.58	0.828
		4–7 h per week	0.63	0.37–1.10	0.104
		> 7 h per week	0.93	0.52–1.64	0.790
	Smoking status	Never/former smoker	Reference		
		Current smoker	1.31	0.57–2.98	0.524

*Note:* Bolded categories indicate a significant *p*‐value when adjusted for multiple comparisons (*p* < 0.002).

Abbreviations: CI, confidence interval; KPS, Karnofsky Performance Status.

**TABLE 5:** Multivariate associations between moderate‐severe REM and demographic and clinical characteristics (*N* = 566).

	Predictor		Full model			Final model		
			Odds ratio	95% CI	*p*‐value	Odds ratio	95% CI	*p*‐value
**Demographic characteristics**	Time from diagnosis		1.00	0.99–1.00	0.084			
	Education				0.642			
		≤ High school education	1.27	0.66–2.46	0.476			
		Any college	0.86	0.47–1.57	0.612			
		Bachelor's degree	0.85	0.53–1.36	0.507			
		Advanced degree	Ref.					
	Grew up in a rural area				0.291			
		No	Ref.					
		Yes	1.27	0.82–1.96	0.291			
**Clinical characteristics**	Top tumor diagnosis				0.188			
		Glioblastoma	Ref.					
		Astrocytoma	1.25	0.69–2.27	0.461			
		Ependymoma	1.07	0.49–2.34	0.870			
		Oligodendroglioma	0.54	0.26–1.13	0.104			
		Other	0.76	0.40–1.47	0.422			
	Tumor grade				0.515			
		Low grade (1/2)	1.21	0.69–2.11	0.515			
		High grade (3/4)	Ref.					
	Number of surgeries				0.141			
		1	Ref.					
		≥ 2	1.45	0.88–2.38	0.141			
	Number of radiation treatment				0.952			
		0	Ref.					
		1	1.07	0.60–1.91	0.829			
		≥ 2	1.15	0.48–2.76	0.754			
	Active treatment at the time of visit				0.608			
		No	Ref.					
		Yes	0.87	0.51–1.48	0.608			
	Progression status				0.130			0.007
		No progression	Ref.			Ref.		
		Progression	1.61	0.87–2.97	0.130	2.02	1.21–3.36	0.007
	Tumor recurrence				0.283			
		No	Ref.					
		Yes	1.36	0.78–2.38	0.283			

Abbreviations: CI, confidence interval; Ref, reference group.

## 4 Discussion

Primary brain tumor patients suffer disproportionally from symptoms and functional limitations resulting from their diagnosis and treatment [6]. In this study, nearly half of the patients reported moderate–severe activity‐related interference or moderate–severe mood‐related interference, with 41% reporting both. This moderate–severe symptom‐related interference has been shown to be caused by clinical factors such as tumor location and number of treatments [8, 9], as well as sociodemographic factors, including social determinants of health such as access to quality healthcare [25]. Among the 28 clinical and sociodemographic factors that were assessed in this study, the number of surgeries, tumor diagnosis, and growing up in a rural area were significant contributors to activity‐related symptom interference, and tumor progression was the primary driver of moderate–severe mood‐related interference in this population.

These findings support that there is a relationship between the extent and intensity of treatment and resulting functional decline [9]. According to prior studies, motor dysfunction in primary brain tumor patients may result from intracranial surgery, thereby impairing a patient's ability to complete their activities of daily living and negatively impacting their functional independence [10]. However, prior studies have not extensively explored this relationship, and our findings revealed that patients with two or more surgeries were more likely to report having moderate–severe activity‐related interference, compared to only having one surgery [38, 39]. Although surgery is generally performed for those with recurrent disease, recurrence alone was not associated with more severe activity‐related interference in multivariate modeling. This may represent differences in those with recurrent disease who are determined to require surgical intervention, such as tumor size, associated edema, or significant symptoms prior to surgery being performed [40].

These findings also support the existence of a relationship between tumor diagnosis and physical function decline, as these outcomes indicated that tumor type significantly contributed to severe activity‐related functional interference. In our study, a diagnosis of ependymoma was a significant driver of moderate–severe activity‐related symptoms, with ependymoma patients having 2.59 times the odds of having moderate–severe activity‐related interference compared to patients with a glioblastoma. The finding in our results that ependymoma tumors contributed to greater symptom severity would appear to contradict prior research in which glioblastoma is known to be the most aggressive in its progression [41, 42], implying that glioblastoma should have the most severe symptoms in comparison to all primary brain tumors. However, glioblastoma survival duration is typically short compared to those of ependymoma, who are more likely to experience additional progression and treatments due to their prolonged survival [41]. Because of this, patients with an ependymoma are forced to adapt to living with a high symptom burden and lasting functional impairments [43, 44]. Further, our results indicated that patients with an ependymoma had greater rates of recurrence (63%) compared to patients with a glioblastoma (49%), in addition to having a greater proportion of patients who had undergone two or more surgeries (52%) compared to that of patients with a glioblastoma (38%). Given what is known about the impact of tumor recurrence as well as multiple surgeries on physical function‐related symptoms, these results may explain why we found patients with ependymomas to be more likely to experience moderate–severe activity‐related interference compared to glioblastoma or other tumor types [45]. Additionally, patients with numerous rounds of treatment and surgery, as seen in patients with ependymomas, also suffer from significant health care costs [46]. As a result, many of these patients are forced to utilize Medicaid, but they are also limited in their access to survivorship treatment such as physical therapy, and as a result, they are less able to address long‐term physical symptoms [44, 47]. This may indicate a reason why patients with ependymomas suffer from greater activity‐related interference compared to those with other tumor types. Lastly, patients with ependymomas were more likely to have tumors of the fourth ventricle (11%) compared to glioblastoma, in which left frontal tumors were more common (4%). Given the location of the fourth ventricle within the brain, these patients may be more likely to suffer from gait or other motor disturbances [48], compared to frontal lobe tumors which are more likely to impact mood and personality [49].

In addition to the impact of clinical factors on interference among primary brain tumor patients, social determinants of health were also found to play a role in interference severity. We have previously reported that residing in a rural area may contribute to mood‐related symptoms [25], but this was expanded upon by our finding that growing up in a rural area contributes to moderate–severe activity‐related interference as well, with these patients having 1.74 times the odds of moderate–severe activity‐related interference compared to patients who grew up in urban areas. As explained by Stockdill and colleagues, where a patient lives may impact their access to healthcare [25], which was confirmed by a separate study in which geographic location was a primary barrier to patients with cancer participating in clinical trials [50]. This may further explain why patients who grew up in rural areas are at a greater risk for activity‐related symptom interference, as access to resources such as clinical trials and quality healthcare are often limited in these areas. Additionally, it has been shown that early life exposures such as diet and physical activity, which may also be impacted by growing up in a rural area, potentially increase cancer risk in adulthood through exposure to risk during critical periods of development in childhood [51]. A study of rural–urban disparities in US cancer survivors also found that rural cancer survivors were more likely to report worse overall health, comorbidities, and greater psychological distress, all of which likely contribute to greater symptom interference [24].

In our analysis of mood‐related symptom interference, we found progression status to be the only significant contributor, with patients experiencing tumor progression having more than two times the likelihood of experiencing moderate–severe mood‐related interference compared to patients that have not had tumor progression. This aligns with current findings in studies of patients with other solid tumor malignancies. For example, a study of patients with breast, lung, and pancreatic cancers noted patients reported that disease progression is related to greater distress not only as a result of the diagnosis or an increase in treatments, but also as a result of the fear of losing control of the cancer and confronting mortality [52]. In the primary brain tumor population, fear and progression status are also closely related, with many patients noting anxiety specifically around disease progression [21]. Others have noted that disease progression may be closely tied to patient depression [53] and quality of life [20]. Additionally, in primary brain tumor patients, cognitive deficits are found to be significantly associated with mood disorders and psychological distress [22]. Cognitive deficits are also known to be one of the harbingers of rapid progression among high‐grade tumors [54], potentially explaining the relationship between progression status and mood‐related symptom interference in this population. In future work, we will investigate whether cognitive deficits associated with tumor progression contribute to moderate–severe mood‐related interference among primary brain tumor patients.

## 5 Conclusion

This study provides a comprehensive look at the clinical and sociodemographic factors that contribute to greater activity and mood‐related interference in the primary brain tumor population in a diverse, cross‐sectional sample.

These findings may be limited by the collection of data at study entry to a natural history study, given participants entered the study at various points in their disease trajectory and evaluation of changes in symptom severity over time was not assessed. This limited our ability to assess the impact of KPS and employment as potential risk factors for activity‐and mood‐related interference, in that the temporality of this association is unknown. Both KPS and employment status were found to be significant individual risk factors; however, they were both excluded from the multivariate modeling because it is unknown whether the changes in levels of interference caused a change in KPS and employment status, or KPS and employment status changed prior to a noted increase in interference levels. In future studies, this relationship should be assessed over time to determine the temporality of this relationship and further explore the association between risk factors and symptom interference. Additionally, in the early life questionnaires utilized at study entry, questions pertaining to family income level while growing up were collected based on the patient's own interpretation of “poor,” “low income,” “middle income,” and “well off,” and therefore, these answers may be subjective based on a patient's own perspective. This study is also limited in that it was initiated at a single, quaternary institution in which referrals are required. This results in limitations that may prevent patients living in rural areas, who are less likely to be referred to specialty centers, from contributing to this study; thus, limiting the diversity of this sample and therefore the widespread application of these findings. Also limiting the diversity of this sample is the small number of racial and ethnically diverse patients, and this should be expanded upon in further studies. As a result of limited sample diversity, there was a smaller number of patients who reported low socioeconomic status compared to the number of patients with higher socioeconomic status. This may have contributed to uneven numbers for variables such as “having enough to eat growing up” (e.g., 31 vs. 426), and therefore, our results show certain non‐significant odds ratios within a potential clinically significant range (e.g., those that did not have enough to eat were over twice as likely to experience mood‐related interference, but this was not statistically significant). Additional sampling of a more diverse patient population in future studies may clarify this relationship. The wide range of tumor diagnoses seen in this sample may also introduce variability in the data, and each primary brain tumor diagnosis should be studied at a greater depth. Given rates of progression tend to vary with different tumor diagnoses, there may be variability in the range of symptoms a patient may experience depending on diagnosis and time since diagnosis. This study evaluated numerous factors contributing to patient symptom burden in a heterogeneous sample of primary brain tumor patients who could enroll in our study at any point in their primary brain tumor trajectory. However, in the future, longitudinal study is necessary to more thoroughly address these differences by tumor type and as specified points of interest in a patient's course of disease. Tumor size and location may also be important contributors to symptom interference, however, this information may be better explored in a more homogenous sample within a longitudinal study. Given the cross‐sectional design of this study in which patients are recruited at different points in the disease process, the impact of tumor size and location is difficult to analyze. Lastly, this study relies on the use of a cutoff score of 2 to differentiate between none‐mild and moderate–severe symptom‐related interference scores. The use of the cutoff score of 2 was based on previous literature, and the cutoff score was implemented to provide more clinically relevant data of patient's symptom‐interference, given moderate–severe symptom interference has been shown to indicate tumor progression in previous studies by our research group and others [11, 32, 33]. However, this cutoff value faces limitations in that previous studies examined cutoff values in cancer patients to distinguish mild to moderate pain, but it has not been thoroughly explored for use in evaluating non‐pain symptoms or within a group of exclusively central nervous system tumor patients. Because of this, future studies should continue evaluating the psychometric properties of this tool by examining the validation of this tool in central nervous system tumor patients, as well as for evaluating non‐pain symptoms related to interference.

This study builds on previous work by assessing a wide range of both clinical and sociodemographic factors in primary brain tumor patients and analyzing this relationship with activity and mood‐related interference. Overall, these analyses demonstrate the significance of both clinical and demographic factors, as well as social determinants of health, on symptom‐related interference in the primary brain tumor population, specifically regarding the role of tumor diagnosis, number of surgeries, growing up in a rural area, and tumor progression. However, it would be beneficial to further explore additional social determinants of health, such as access to health care, housing, and economic stability, which were not assessed in this report. An improved understanding of the risk factors for greater symptom interference may be used to assist providers in identifying patients most at risk for activity and mood‐related symptom interference, as well as to further explore the changes in symptoms and clinical and sociodemographic risk factors over time to develop more effective interventions.

## Author Contributions

**Bennett A. McIver:** conceptualization (equal), investigation (equal), visualization (lead), writing – original draft (lead). **Tara S. Davis:** investigation (supporting), writing – review and editing (supporting). **Kimberly Reinhart:** data curation (lead), formal analysis (lead), methodology (supporting), writing – original draft (supporting), writing – review and editing (supporting). **Elizabeth Vera:** data curation (equal), formal analysis (equal), methodology (lead), supervision (equal), writing – review and editing (supporting). **Alvina Acquaye‐Mallory:** investigation (equal), project administration (equal), writing – review and editing (supporting). **Anna Choi:** investigation (equal), project administration (equal), writing – review and editing (supporting). **Tricia Kunst:** investigation (equal), project administration (equal), writing – review and editing (supporting). **Morgan Johnson:** investigation (equal), writing – review and editing (supporting). **Ewa Grajkowska:** data curation (equal), project administration (equal), writing – review and editing (supporting). **Hope Miller:** data curation (equal), project administration (equal), writing – review and editing (supporting). **Jennifer Reyes:** project administration (equal), writing – review and editing (supporting). **Mark R. Gilbert:** investigation (equal), resources (equal), writing – review and editing (supporting). **Terri S. Armstrong:** conceptualization (equal), funding acquisition (lead), resources (equal), writing – review and editing (equal). **Michelle L. Wright:** conceptualization (equal), investigation (equal), supervision (equal), writing – review and editing (equal).

## Conflicts of Interest

The authors declare no conflicts of interest.

## Supporting information


Data S1.
**Figure S1**. Activity (WAW) and Mood‐related (REM) interference scores by sex.
**Figure S2**. Activity (WAW) and Mood‐related (REM) interference scores by ethnicity.
**Figure S3**. Activity (WAW) and Mood‐related (REM) interference scores by rural area.
**Figure S4**. Activity (WAW) and Mood‐related (REM) interference scores by enough to eat growing up.
**Figure S5**. Activity (WAW) and Mood‐related (REM) interference scores by progression status.
**Figure S6**. Activity (WAW) and Mood‐related (REM) interference scores by race.
**Figure S7**. Activity (WAW) and Mood‐related (REM) interference scores by education status.
**Figure S8**. Activity (WAW) and Mood‐related (REM) interference scores by employment status.
**Figure S9**. Activity (WAW) and Mood‐related (REM) interference scores by income categories.
**Figure S10**. Activity (WAW) and Mood‐related (REM) interference scores by family income level.
**Figure S11**. Activity (WAW) and Mood‐related (REM) interference scores by tumor diagnosis.
**Figure S12**. Activity (WAW) and Mood‐related (REM) interference scores by number of surgeries.

## Data Availability

The data that support the findings of this study are available on request from the corresponding author. The data are not publicly available due to restrictions (e.g., their containing information that could compromise the privacy of research participants).
