# Supplementary material for: Evaluating Clinical and Sociodemographic Risk for Symptom Burden Associated Interference With Daily Functioning in the Primary Brain Tumor Patient Population
Source: Cancer Med. 2025 Mar 7;14(5):e70682. doi: 10.1002/cam4.70682 (PMC11886885; doi:10.1002/cam4.70682)

Supporting Information Figure 1:

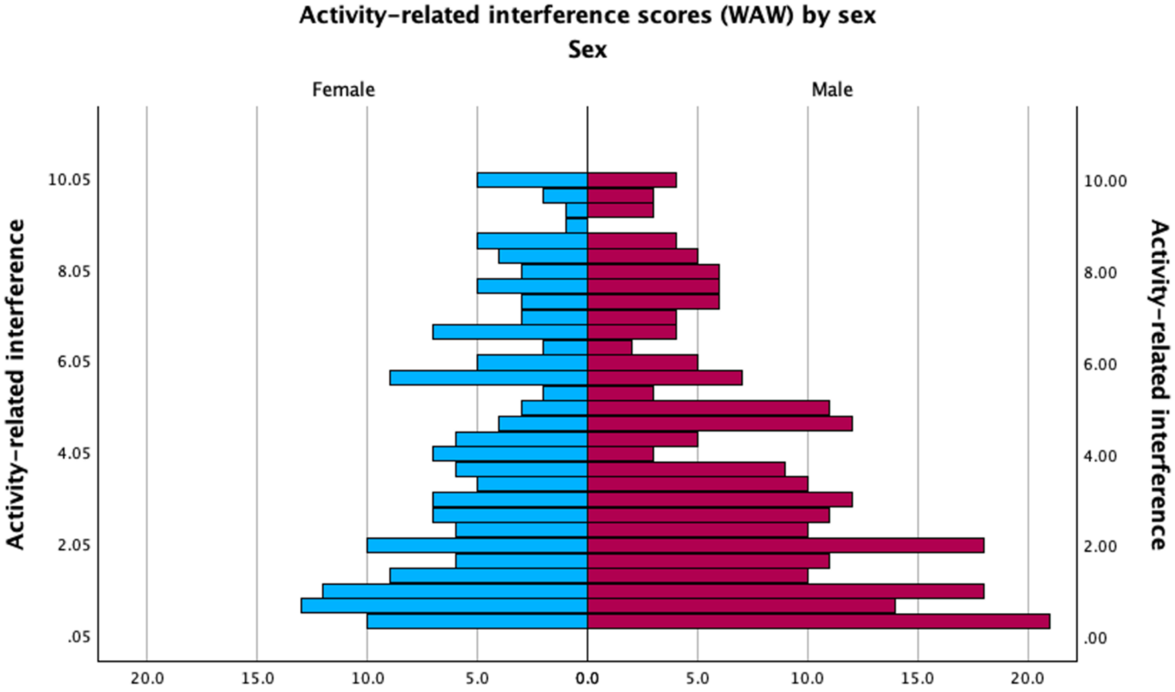

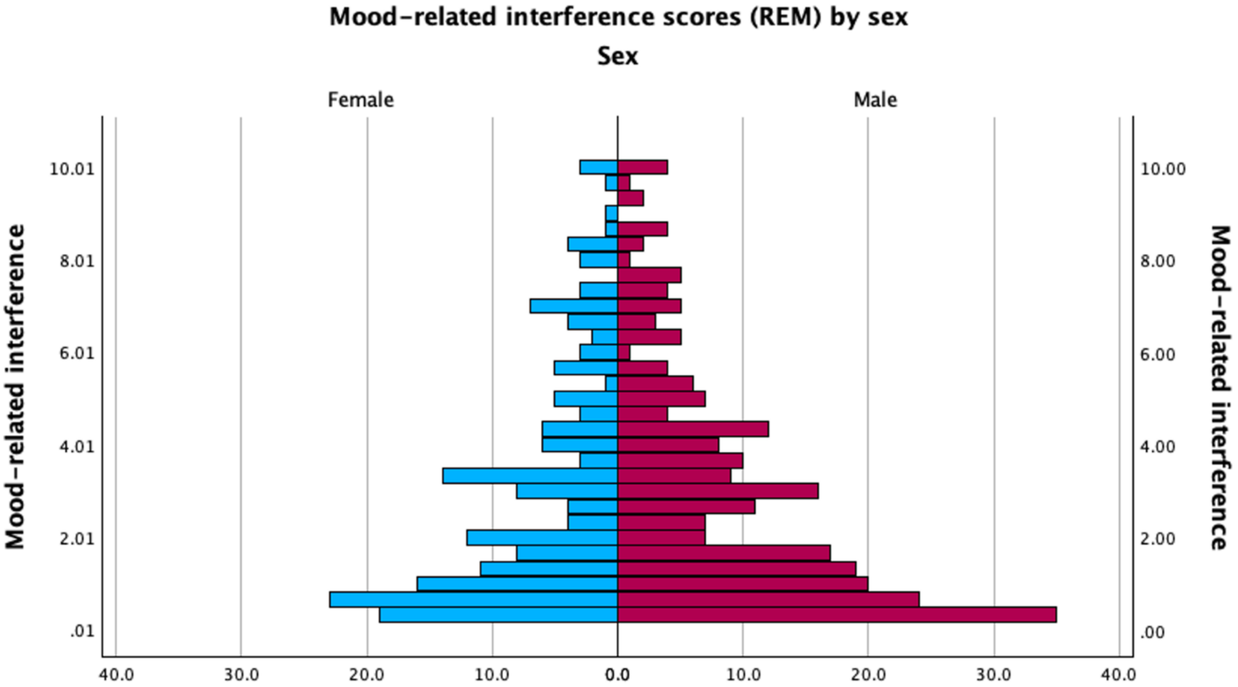

Only patients with interference scores above 0 were plotted in each figure.

Supporting Information Figure 2:

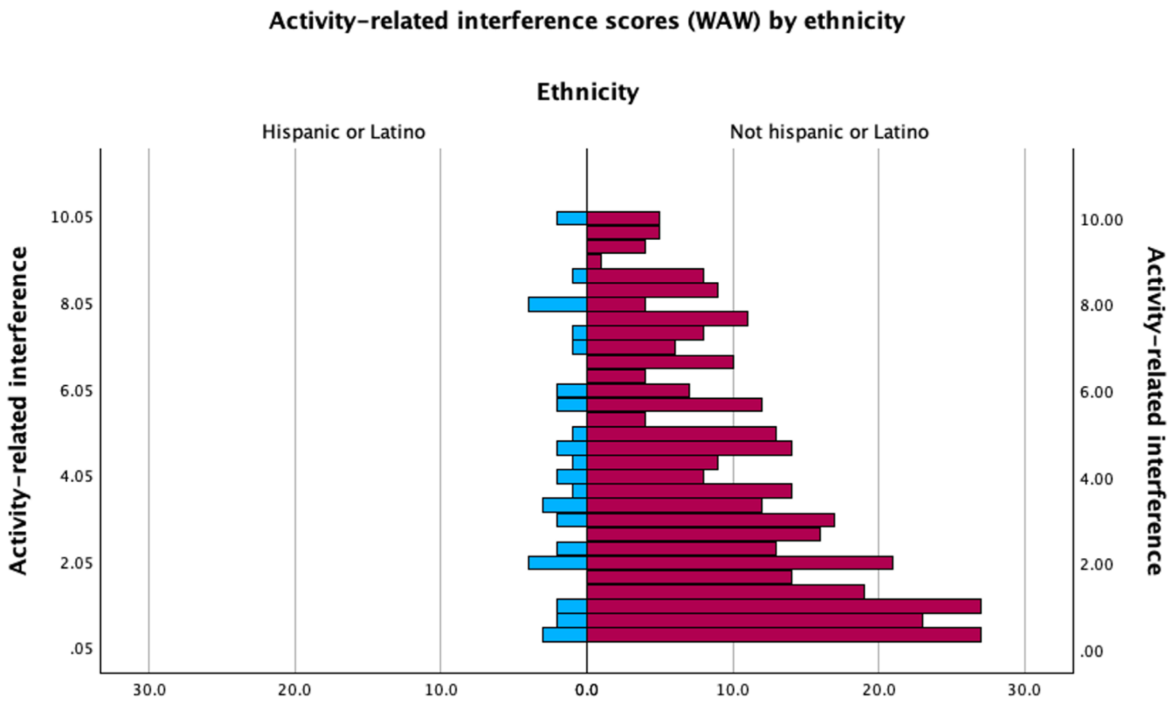

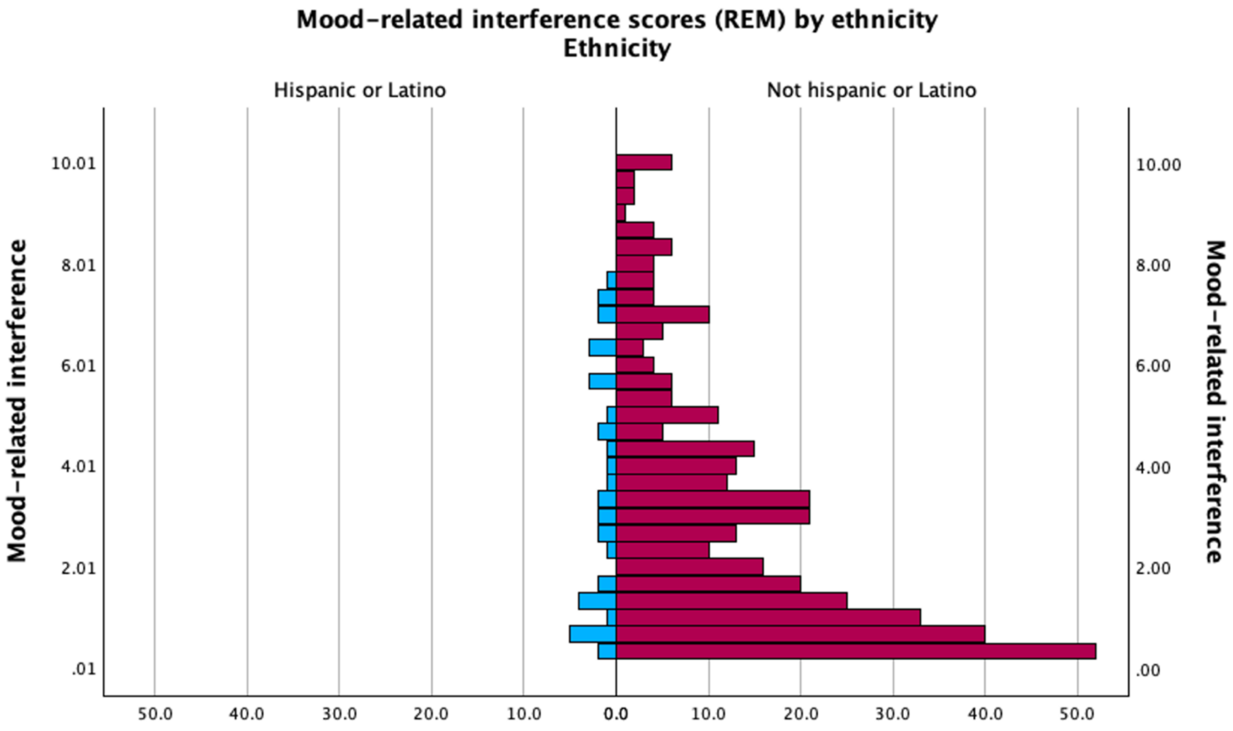

Only patients with interference scores above 0 were plotted in each figure.

Supporting Information Figure 3:

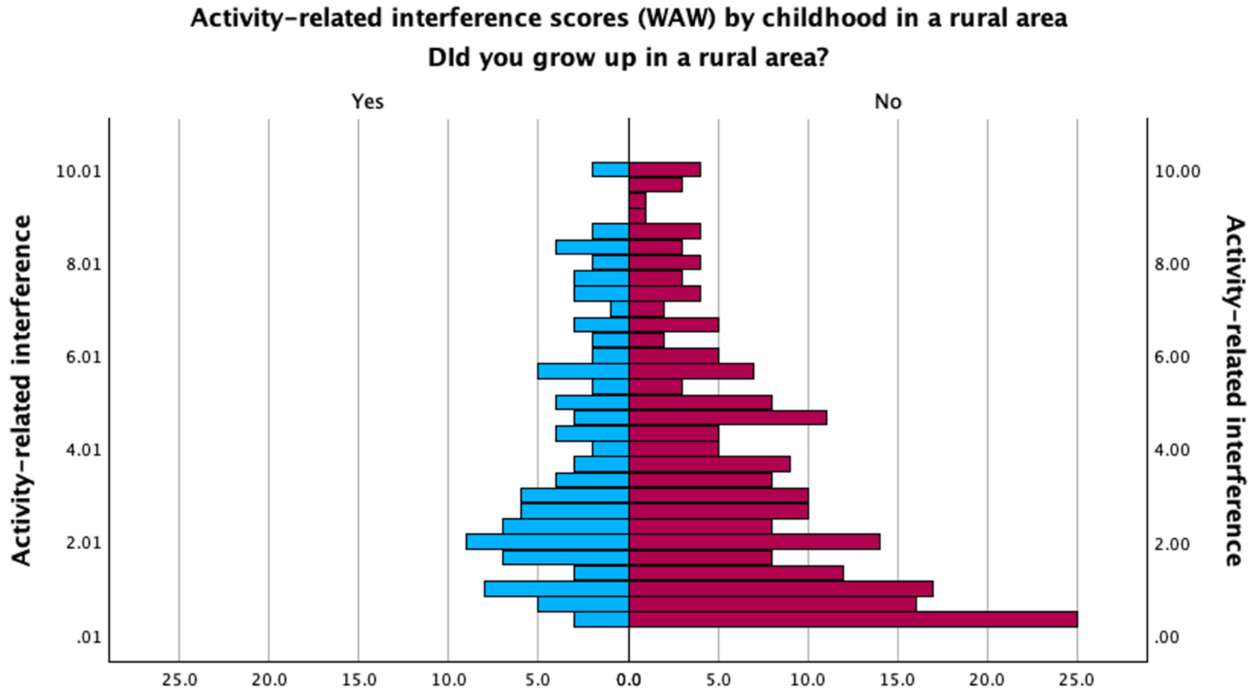

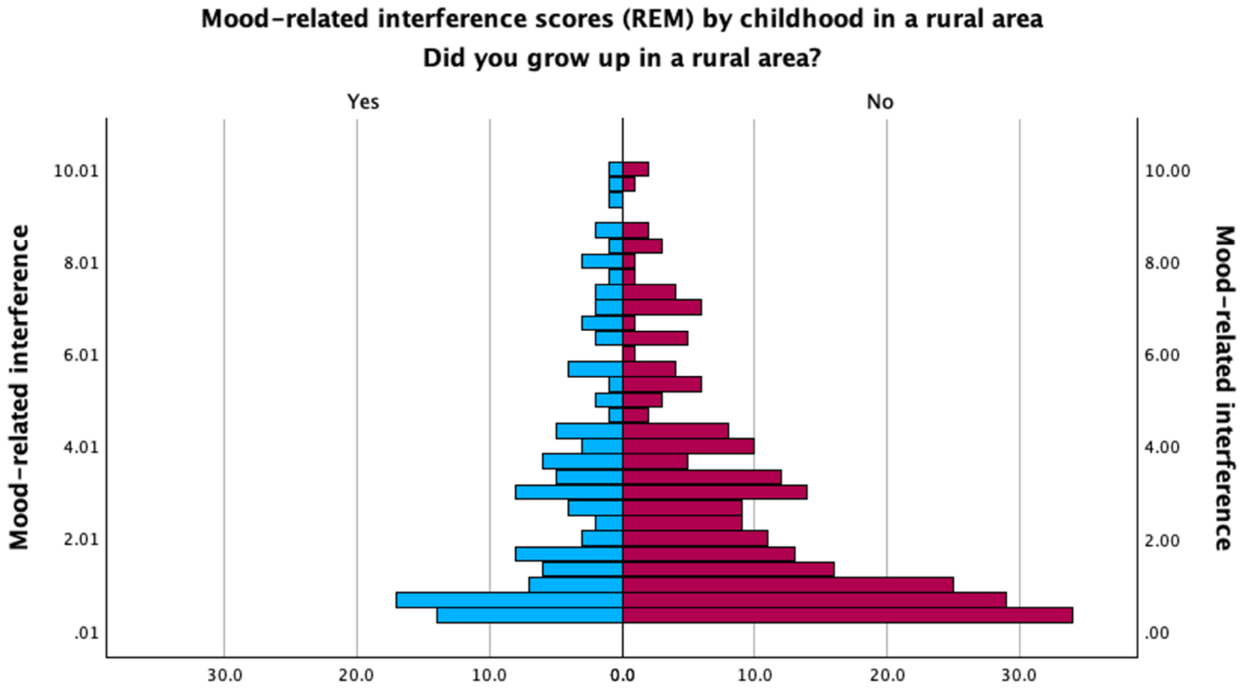

Only patients with interference scores above 0 were plotted in each figure.

Supporting Information Figure 4:

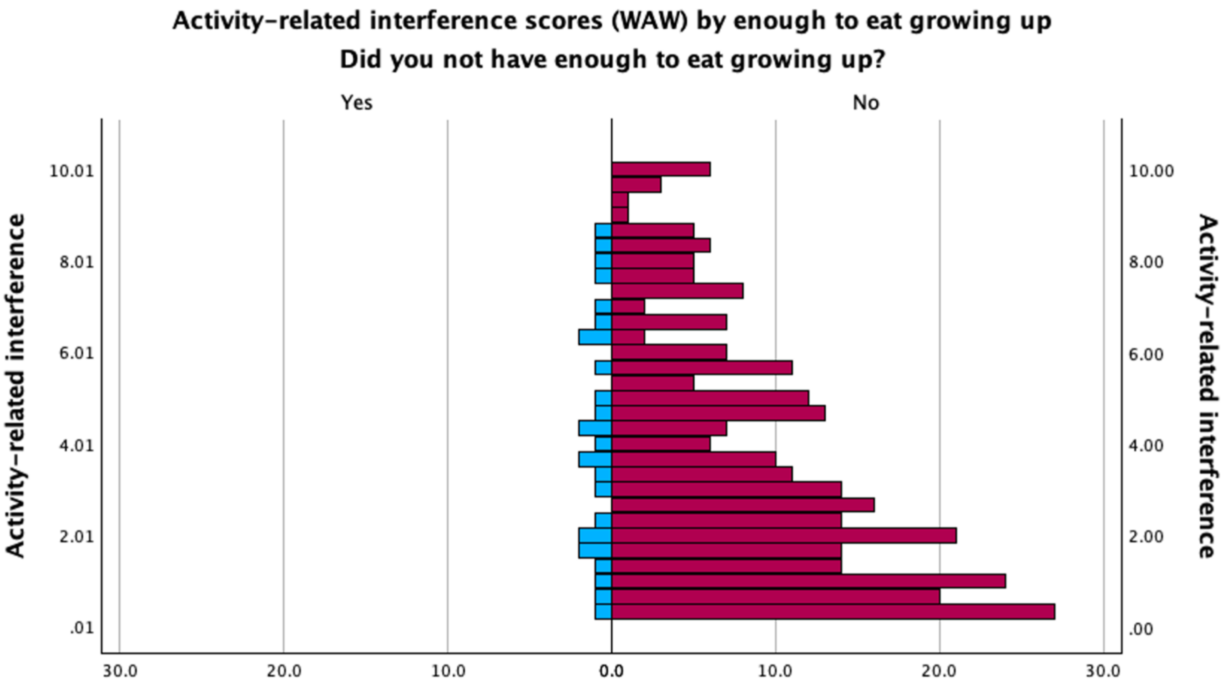

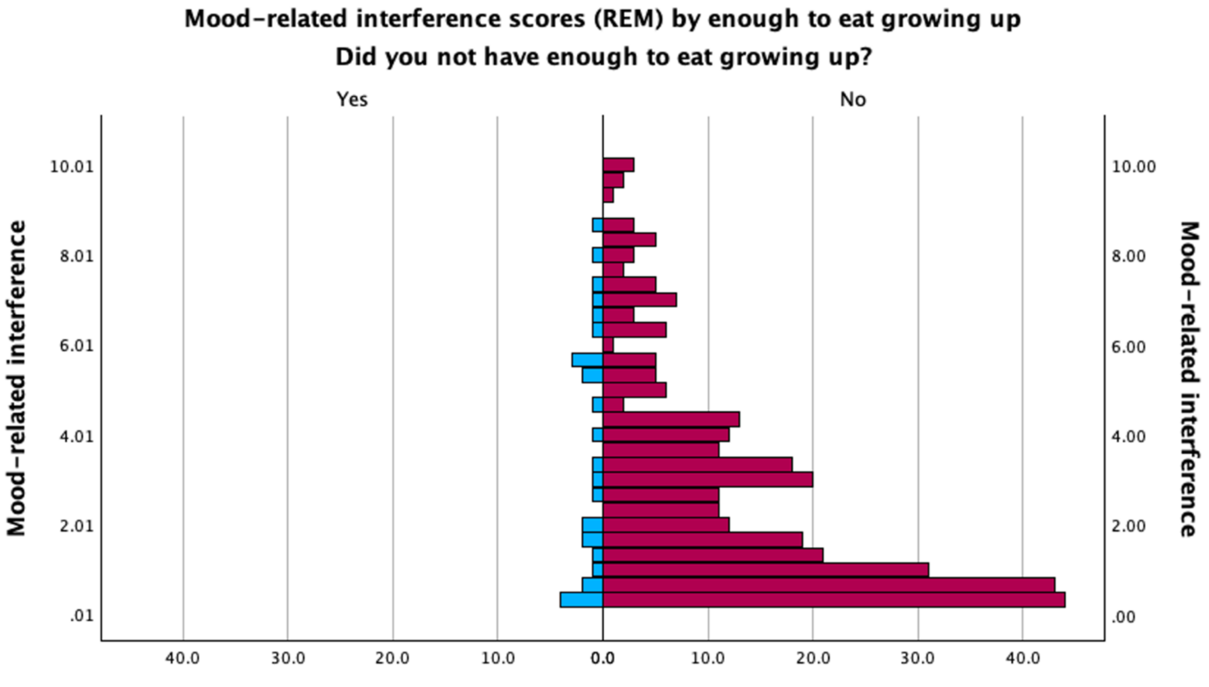

Only patients with interference scores above 0 were plotted in each figure.

Supporting Information Figure 5:

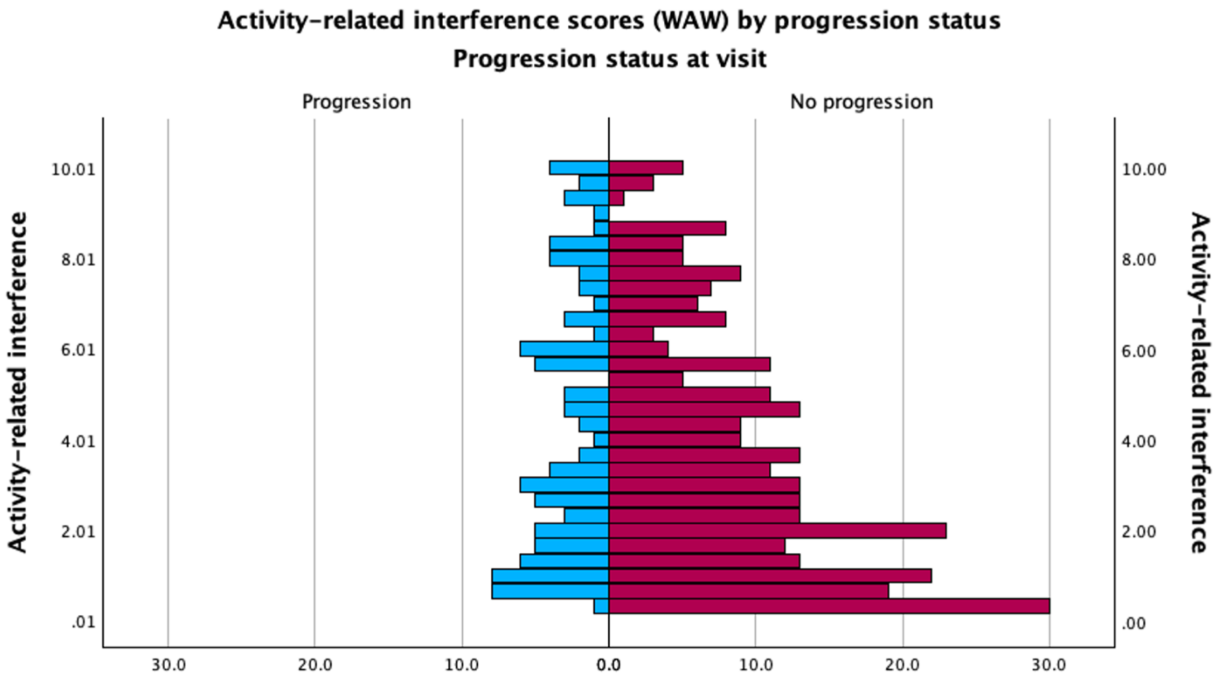

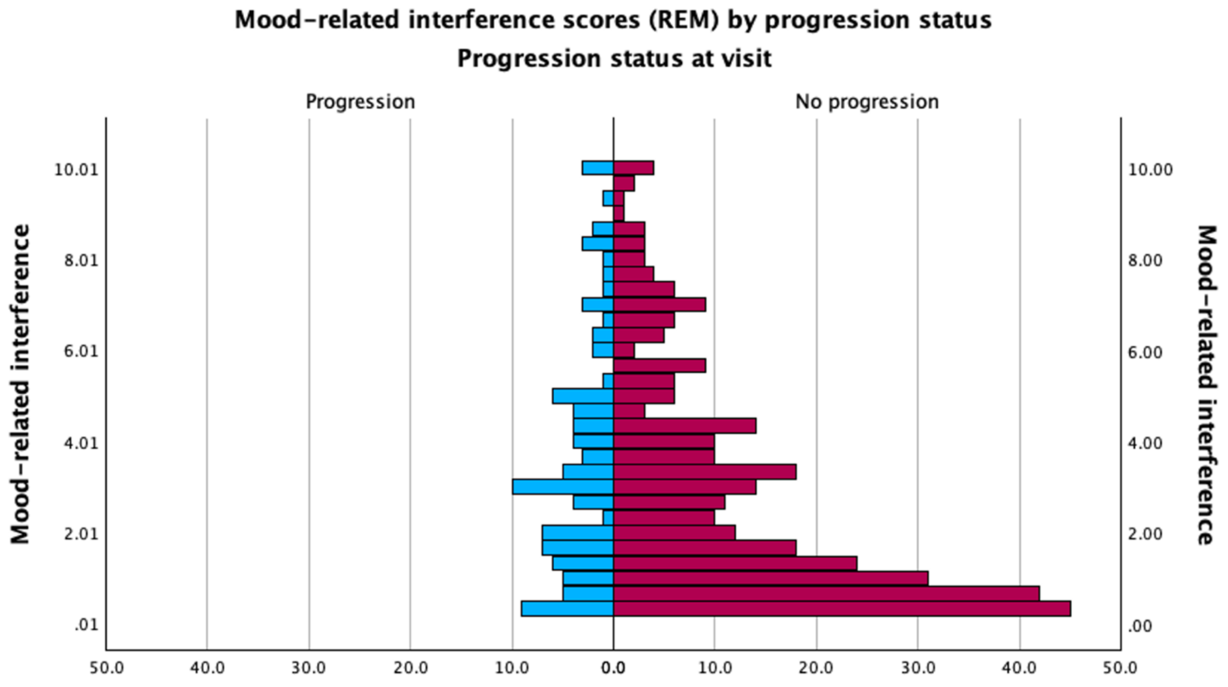

Only patients with interference scores above 0 were plotted in each figure.

Supporting Information Figure 6:

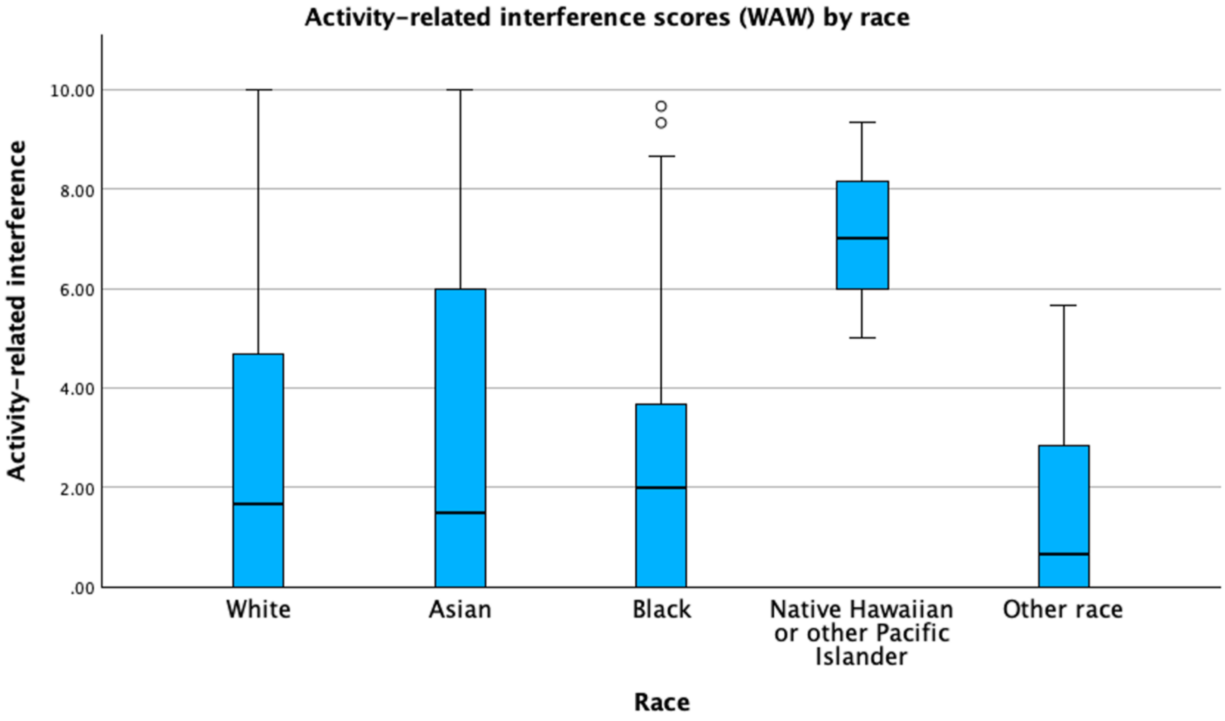

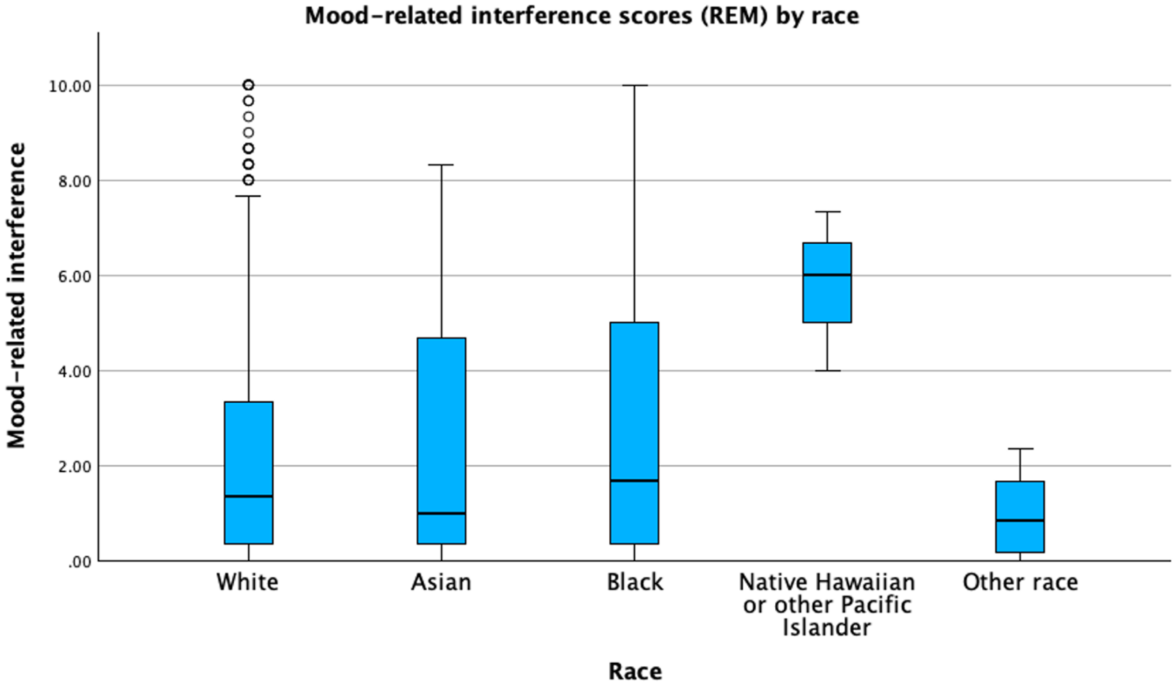

Supporting Information Figure 7:

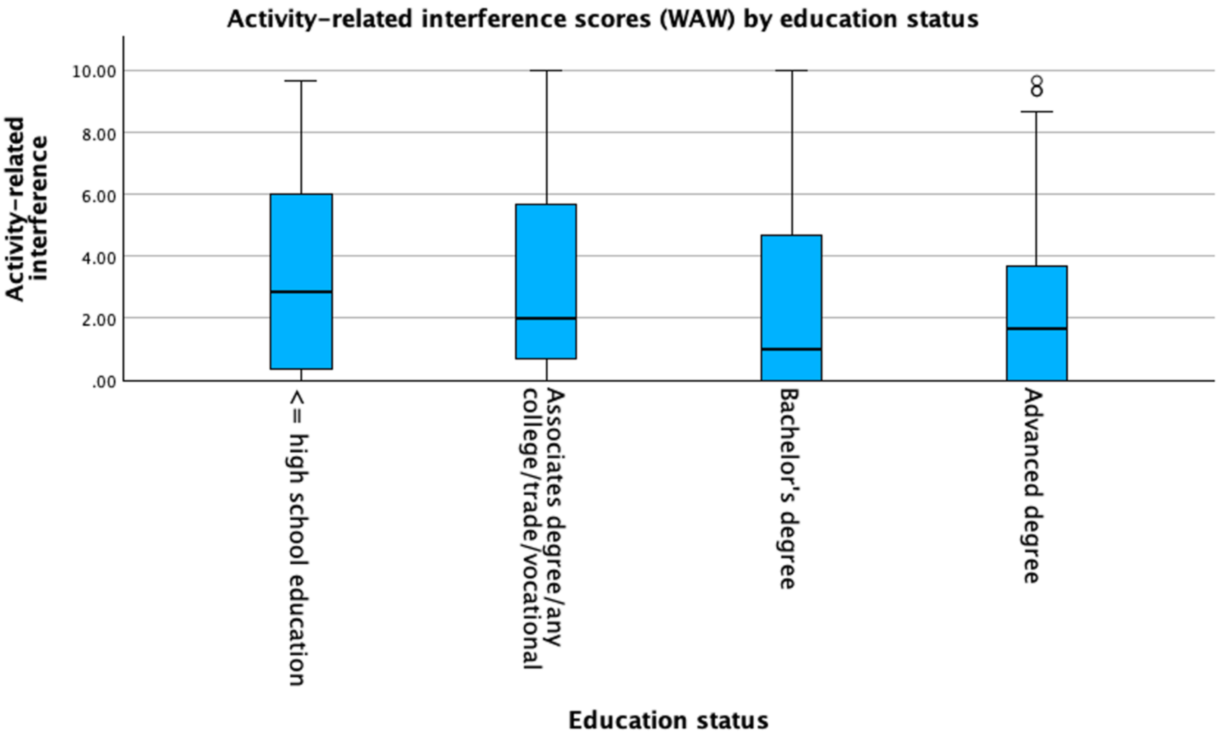

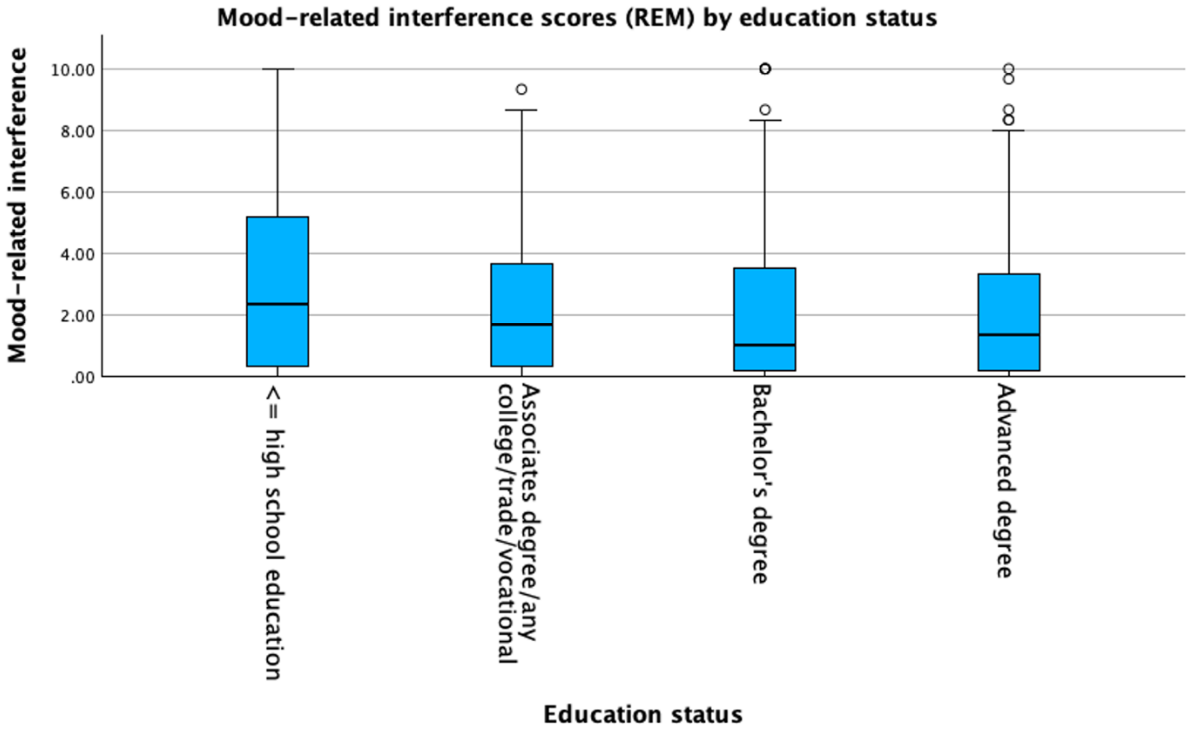

Supporting Information Figure 8:

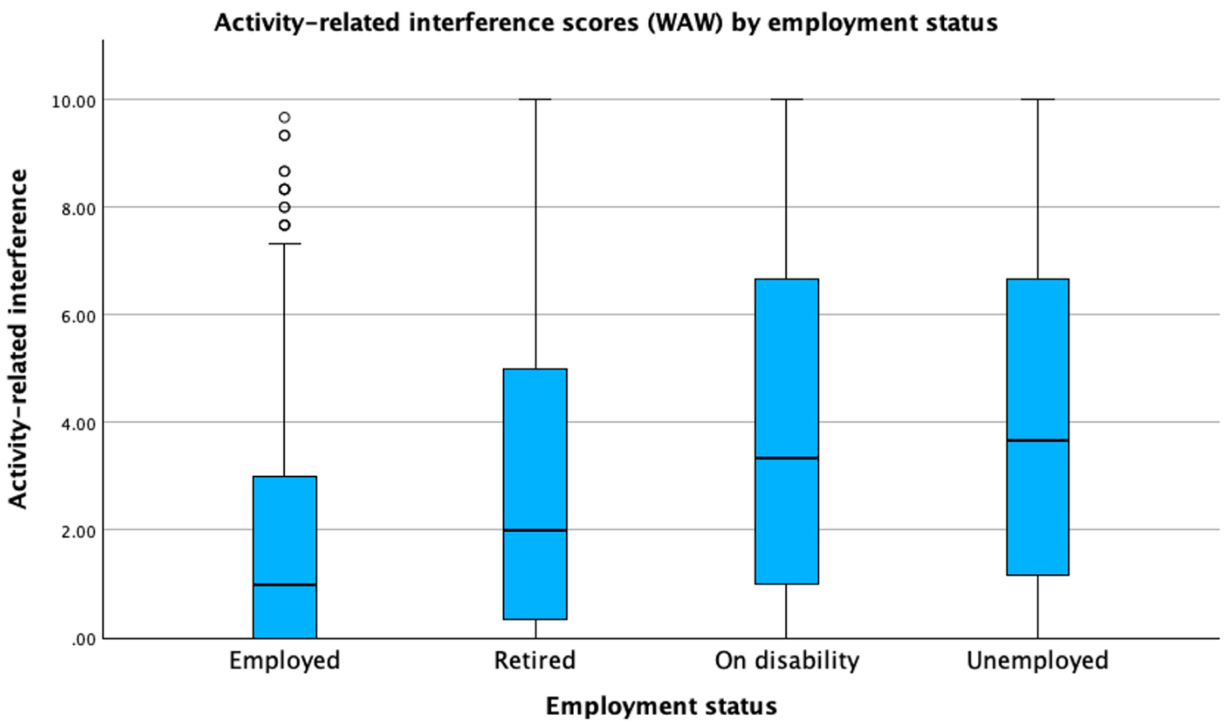

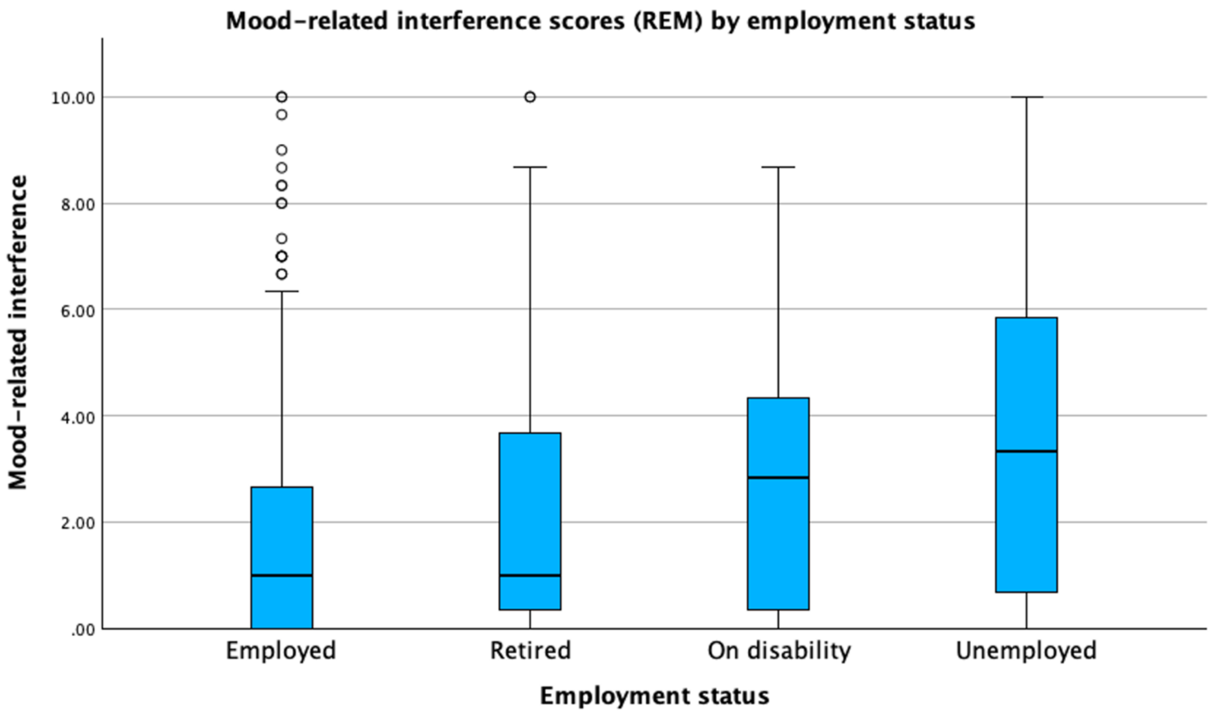

Supporting Information Figure 9:

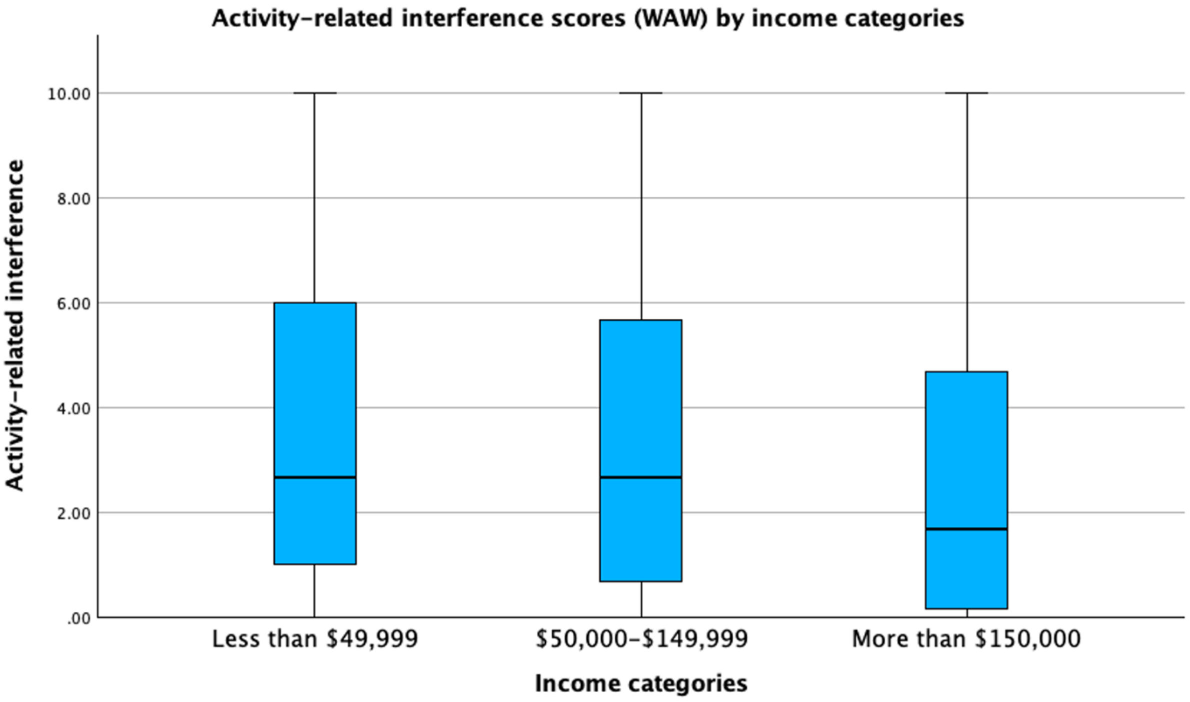

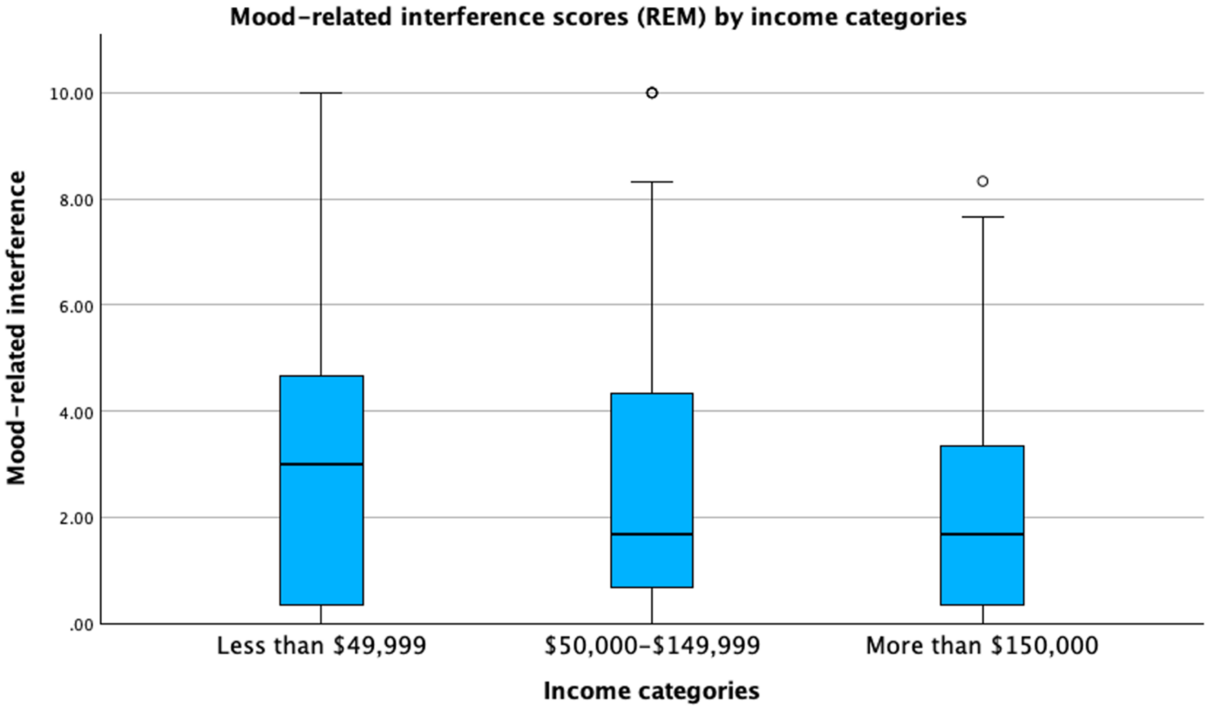

Supporting Information Figure 10:

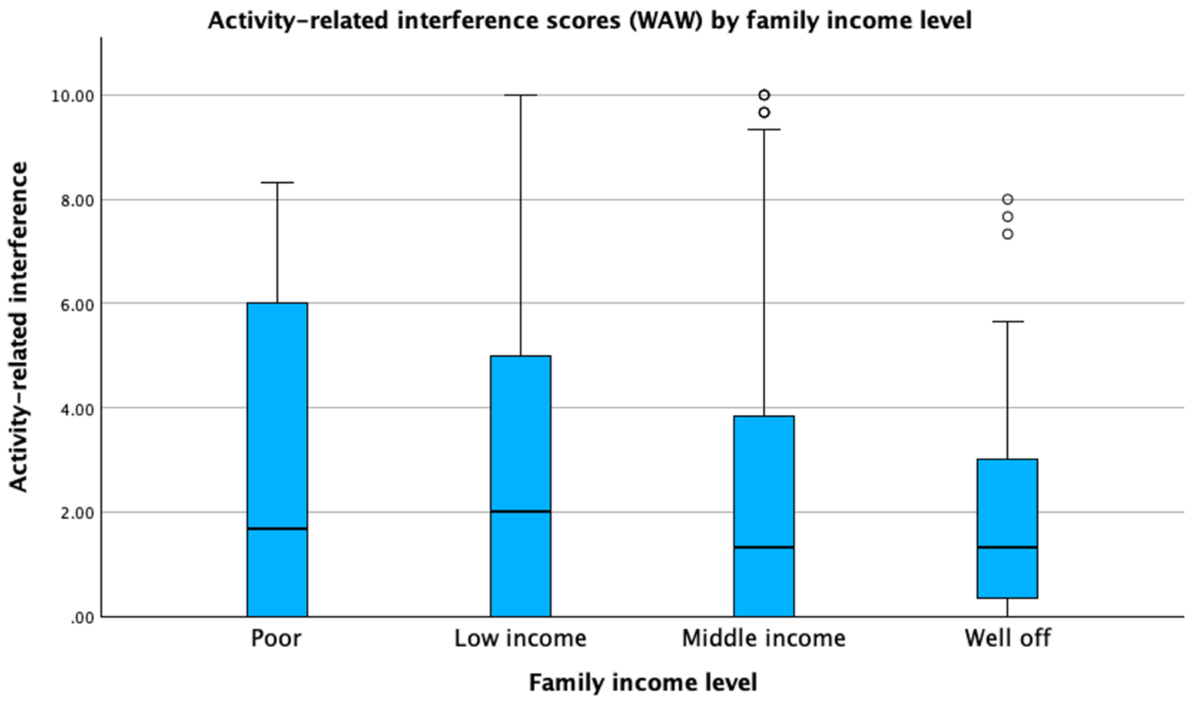

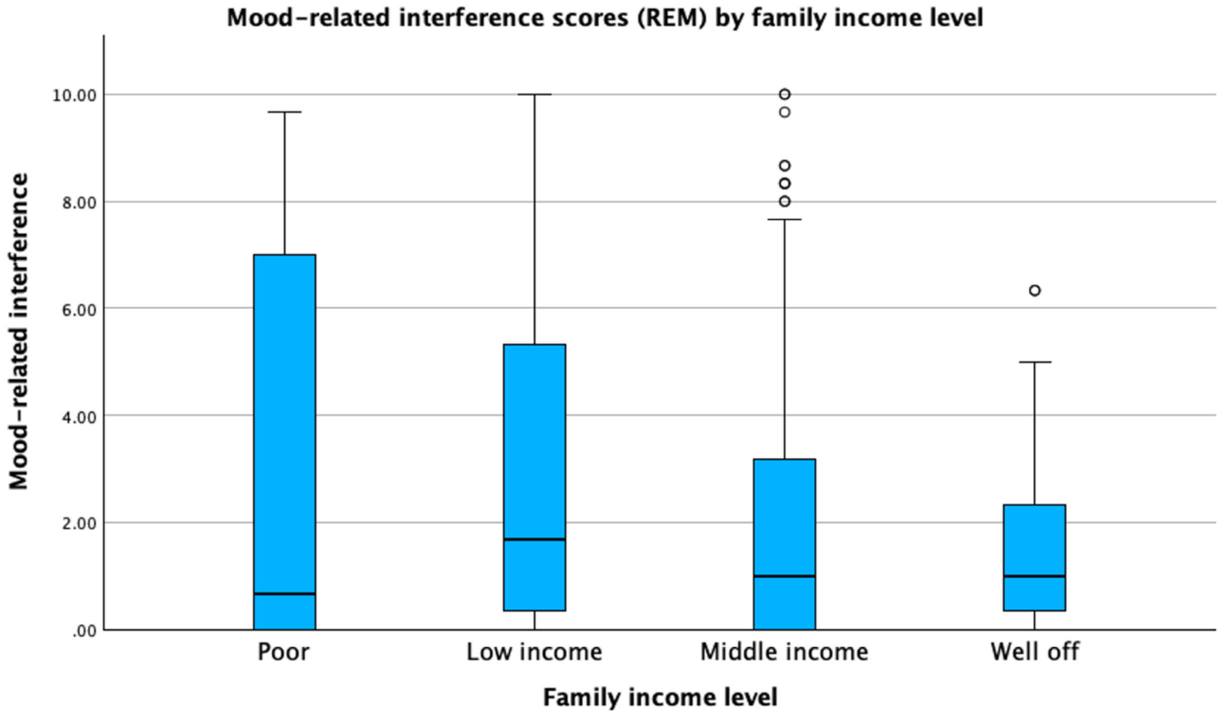

Supporting Information Figure 11:

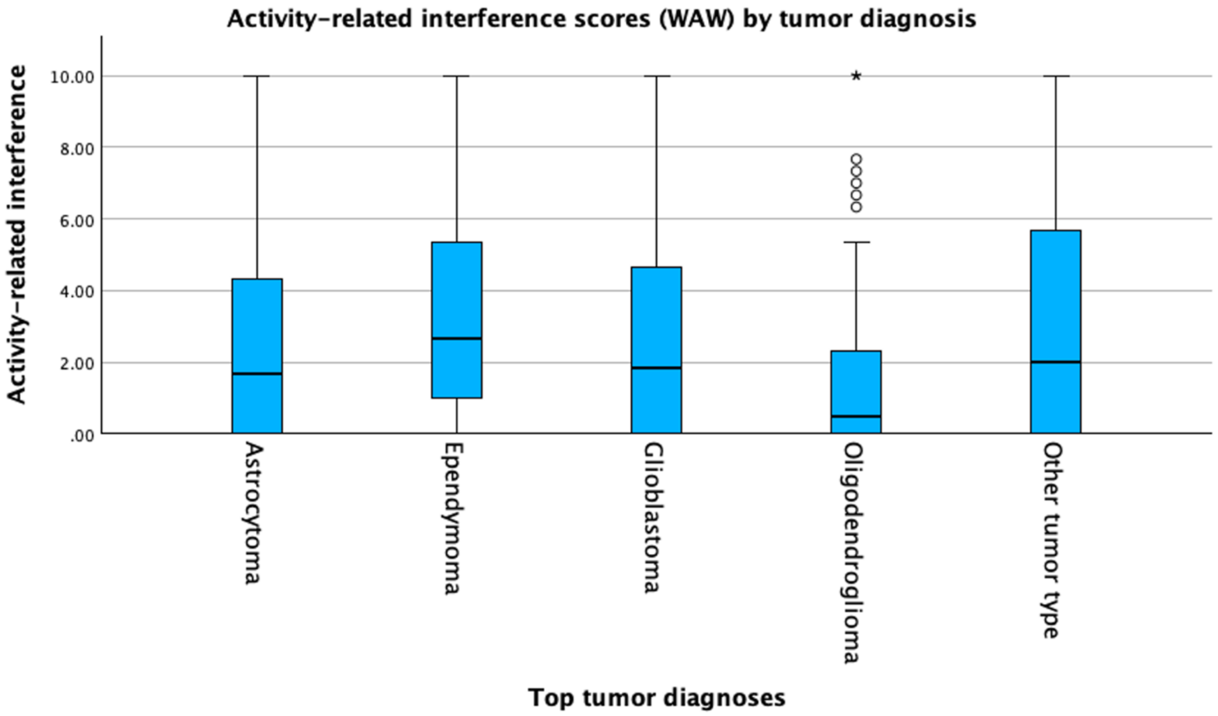

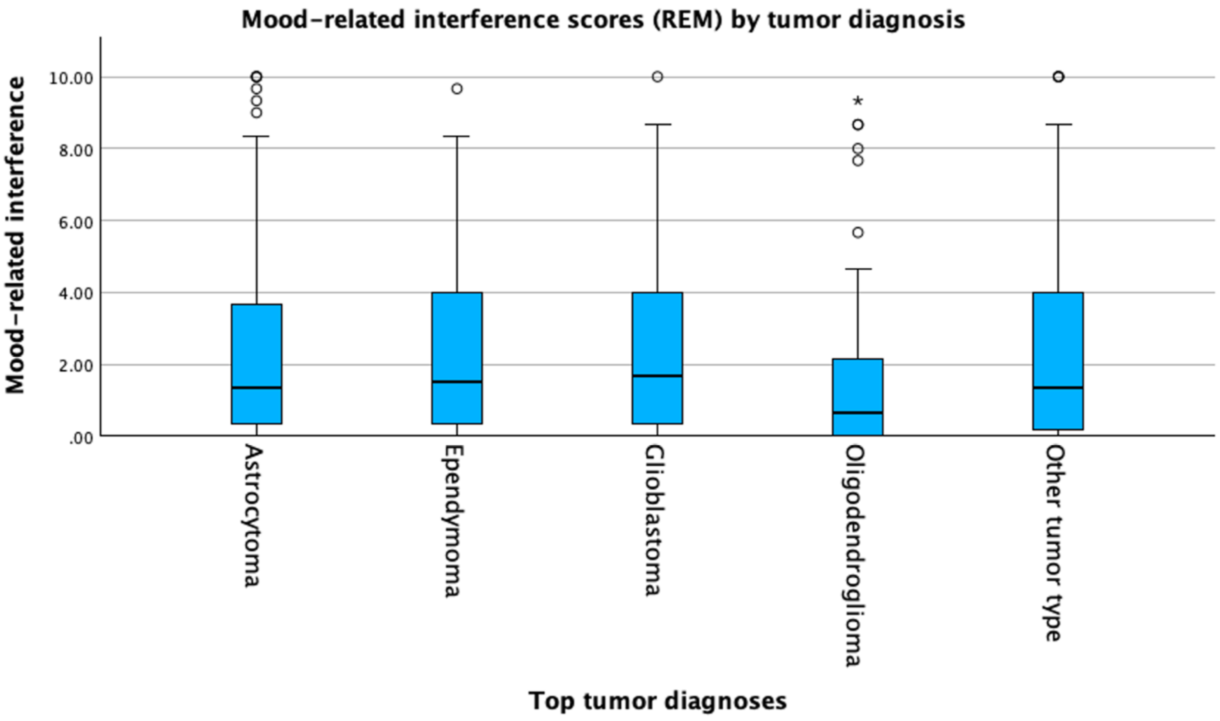

Supporting Information Figure 12:

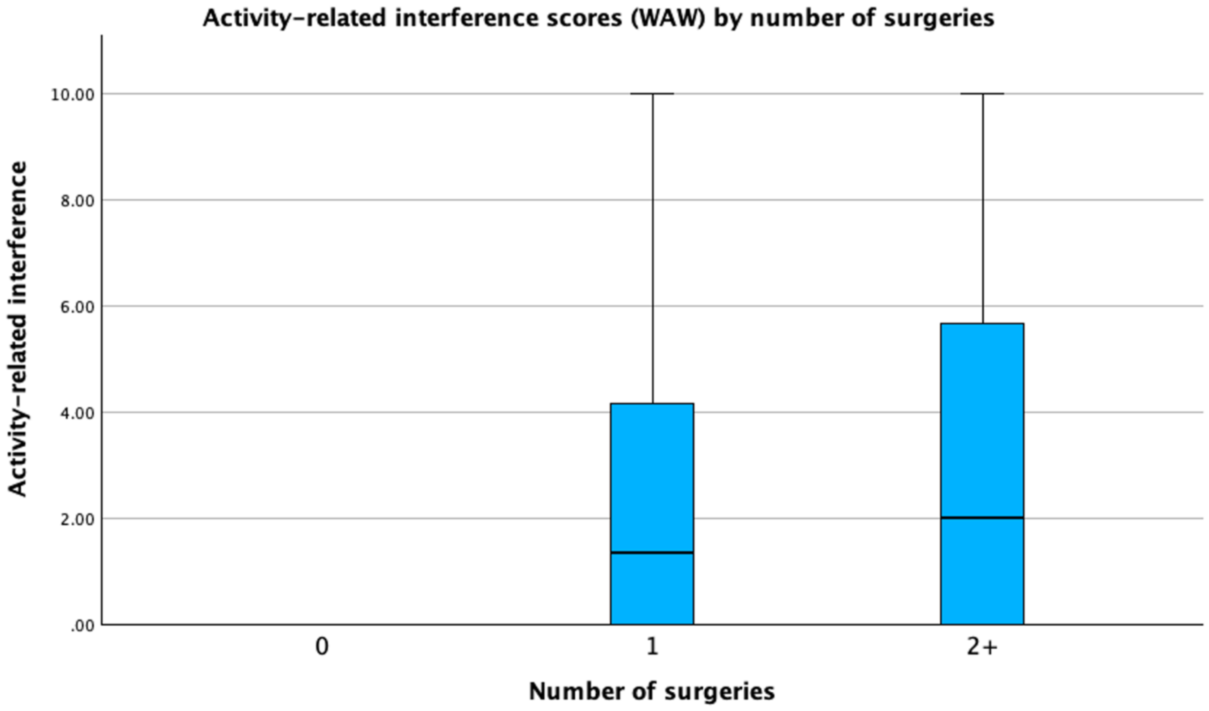

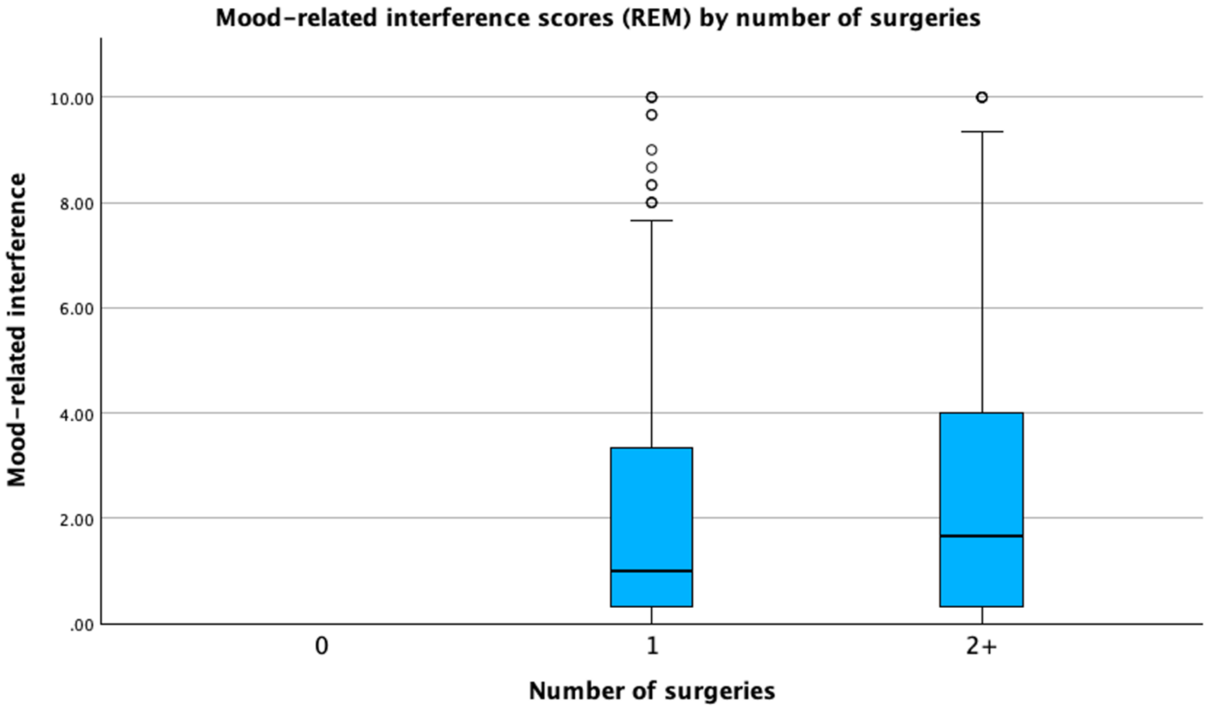

Supplement: Supplementary file 1 — Data S1. Figure S1. Activity (WAW) and Mood‐related (REM) interference scores by sex. Figure S2. Activity (WAW) and Mood‐related (REM) interference scores by ethnicity. Figure S3. Activity (WAW) and Mood‐related (REM) interference scores by rural area. Figure S4. Activity (WAW) and Mood‐related (REM) interference scores by enough to eat growing up. Figure S5. Activity (WAW) and Mood‐related (REM) interference scores by progression status. Figure S6. Activity (WAW) and Mood‐related (REM) interference scores by race. Figure S7. Activity (WAW) and Mood‐related (REM) interference scores by education status. Figure S8. Activity (WAW) and Mood‐related (REM) interference scores by employment status. Figure S9. Activity (WAW) and Mood‐related (REM) interference scores by income categories. Figure S10. Activity (WAW) and Mood‐related (REM) interference scores by family income level. Figure S11. Activity (WAW) and Mood‐related (REM) interference scores by tumor diagnosis. Figure S12. Activity (WAW) and Mood‐related (REM) interference scores by number of surgeries. [file CAM4-14-e70682-s001.pdf]
